# TrkB Agonist (7,8-DHF)-Induced Responses in Dorsal Root Ganglia Neurons Are Decreased after Spinal Cord Injury: Implication for Peripheral Pain Mechanisms

**DOI:** 10.1523/ENEURO.0219-24.2024

**Published:** 2025-01-03

**Authors:** Kyeongran Jang, Sandra M. Garraway

**Affiliations:** Department of Cell Biology, School of Medicine, Emory University, Atlanta, Georgia 30322

**Keywords:** DRG, pain, peripheral, sensory neuron, spinal cord injury, TrkB signaling

## Abstract

Brain-derived neurotrophic factor (BDNF) and tropomyosin receptor kinase B (TrkB) are known to contribute to both protective and pronociceptive processes. However, their contribution to neuropathic pain after spinal cord injury (SCI) needs further investigation. In a recent study utilizing TrkB^F616A^ mice, it was shown that systemic pharmacogenetic inhibition of TrkB signaling with 1NM-PP1 (1NMP) immediately after SCI delayed the onset of pain hypersensitivity, implicating maladaptive TrkB signaling in pain after SCI. To examine potential neural mechanisms underlying the behavioral outcome, patch-clamp recording was performed in small-diameter dissociated thoracic (T) dorsal root ganglia (DRG) neurons to evaluate TrkB signaling in uninjured mice and after T10 contusion SCI. Bath-applied 7,8-dihydroxyflavone (7,8-DHF), a selective TrkB agonist, induced a robust inward current in neurons from uninjured mice, which was attenuated by 1NMP treatment. SCI also decreased 7,8-DHF-induced current while increasing the latency to its peak amplitude. Western blot revealed a concomitant decrease in TrkB expression in DRGs adjacent to the spinal lesion. Analyses of cellular and membrane properties showed that SCI increased neuronal excitability, evident by an increase in resting membrane potential and the number of spiking neurons. However, SCI did not increase spontaneous firing in DRG neurons. These results suggest that SCI induced changes in TrkB activation in DRG neurons even though these alterations are likely not contributing to pain hypersensitivity by nociceptor hyperexcitability. Overall, this reveals complex interactions involving TrkB signaling and provides an opportunity to investigate other, presumably peripheral, mechanisms by which TrkB contributes to pain hypersensitivity after SCI.

## Significance Statement

TrkB signaling is linked to both adaptive and maladaptive plasticity including neuropathic pain after SCI. This study investigated TrkB signaling in DRG neurons as a potential neural mechanism that underlies pain hypersensitivity. The results revealed that TrkB-mediated inward current and its protein expression are drastically reduced in the DRG after SCI. Furthermore, SCI altered several neuronal properties, consistent with increased neuronal hyperexcitability, although not exclusively in a TrkB-dependent manner. Together, these results reveal that although TrkB signaling in DRG neurons might not underlie nociceptor hyperexcitability or neuropathic pain, more specific targeting of TrkB mechanisms, including peripheral components, is needed to address SCI-induced pain.

## Introduction

Neuropathic pain, a pain caused by damage to the nervous system, is a common consequence of spinal cord injury (SCI; Siddall and Loeser, 2001; Felix et al., 2022). The mechanisms underlying neuropathic pain are complex and shown to involve both central and peripheral mediators (see reviews by Hulsebosch, 2002; Hulsebosch et al., 2009). Brain-derived neurotrophic factor (BDNF), signaling through its high affinity tropomyosin receptor kinase-B (TrkB), serves as an effective growth promoter, including regrowth of damaged axons in the injured spinal cord. However, BDNF is also a potent modulator of pain signaling. BDNF is expressed and synthesized in small-to-medium diameter dorsal root ganglia (DRG) neurons (Kerr et al., 1999; Obata and Noguchi, 2006; Merighi et al., 2008), and its levels are altered in the DRG and spinal cord dorsal horn after tissue or nerve injury (Ernfors et al., 1993; Michael et al., 1997; Cho et al., 1998; Zhou et al., 1999; Fukuoka et al., 2001; Ha et al., 2001; Pezet et al., 2002; Merighi et al., 2008). TrkB is also expressed in DRGs (Hougland et al., 2012; Y. Zheng et al., 2019), and its activation is linked with hypersensitization of thermal sensory neurons (Shu et al., 1999) and mechanical allodynia (Dhandapani et al., 2018; Hu et al., 2023). The duplicity of BDNF-TrkB signaling in both the peripheral and central nervous systems demonstrate the complex involvement of the neurotrophin system and underscores the need for deeper investigation into its mechanistic contribution in postinjury plasticity. Especially after SCI, the role of TrkB activation in generation or maintenance of pain hypersensitivity has not been fully elucidated (see review by Garraway and Huie 2016).

A recent study showed that pharmacogenetic inhibition of TrkB signaling immediately after SCI delayed the onset of mechanical hypersensitivity and improved locomotor recovery in adult TrkB^F616A^ mice (F616; Martin et al., 2022). Inhibition of TrkB at later time points also reversibly attenuated mechanical hypersensitivity. F616 mice express TrkB receptors containing a mutation that allows a small molecule, cell-permeable kinase inhibitor, 1NM-PP1 (1NMP), to bind to the ATP-binding pocket (X. Chen et al., 2005). 1NMP provides a rapid (typically within an hour in vivo; Pareja-Cajiao et al. 2020), robust, and reversible inhibition of TrkB (X. Chen et al., 2005). Until this study, research had suggested a minimal role of spinal BDNF or TrkB in SCI-induced pain. For example, BDNF and TrkB levels decreased in the injured spinal cord (King et al., 2000; Liebl et al., 2001; Hajebrahimi et al., 2008; Garraway et al., 2011; Strickland et al., 2014) even when mechanical hypersensitivity was evident (Garraway et al., 2011). Moreover, BDNF failed to induce LTP-like facilitation of C-fiber evoked synaptic and glutamatergic responses after SCI (Garraway et al., 2005; Garraway and Mendell, 2007), unlike its facilitatory actions in uninjured rats (Garraway et al., 2005). No study has directly assessed whether TrkB signaling in pain after SCI is mediated by spinal TrkB activation or changes in TrkB elsewhere, although in inflammatory and neuropathic pain models, peripheral BDNF was found to be involved in mediating transition from acute to chronic pain (Sikandar et al., 2018). The potential contribution of peripheral BDNF and TrkB to plasticity after SCI has not been fully explored, and the recent observation that maladaptive TrkB signaling contributes to mechanical pain acutely after SCI (Martin et al., 2022) suggests a more complex role for TrkB signaling in nociceptive plasticity. However, the specific neural mechanisms, including the exact site of action, were not determined. Building upon this finding, this study proposes that the engagement of TrkB mechanisms drives changes in excitability of DRG neurons after SCI, postulating that peripheral TrkB mechanisms underlie pain.

DRGs, uniquely positioned at the intersection between the peripheral and central nervous system, play a key role in nociceptive transmission. Bedi et al. (2010) found primary nociceptors to become chronically hyperexcitable after SCI, consistent with pain hypersensitivity. Here, dissociated DRG neurons obtained from uninjured or SCI F616 mice, treated with 1NMP or vehicle, were evaluated using whole-cell patch-clamp electrophysiology. The study investigated neuronal responses to 7,8-dihydroxyflavone (7,8-DHF), a small molecule, selective TrkB agonist that binds TrkB with more specificity than BDNF but triggers the same downstream intracellular signaling cascade (S-W. Jang et al., 2010). Furthermore, changes in intrinsic properties of sensory neurons, capsaicin-induced inward currents, and TrkB protein expression in DRGs were assessed. Overall, the results revealed that SCI induced neuronal hyperexcitability, partly independent of TrkB signaling, and that both TrkB agonist-induced neuronal responses and TrkB expression in DRGs adjacent to the level of injury were reduced after SCI.

## Materials and Methods

### Subjects

**Figure 1.**
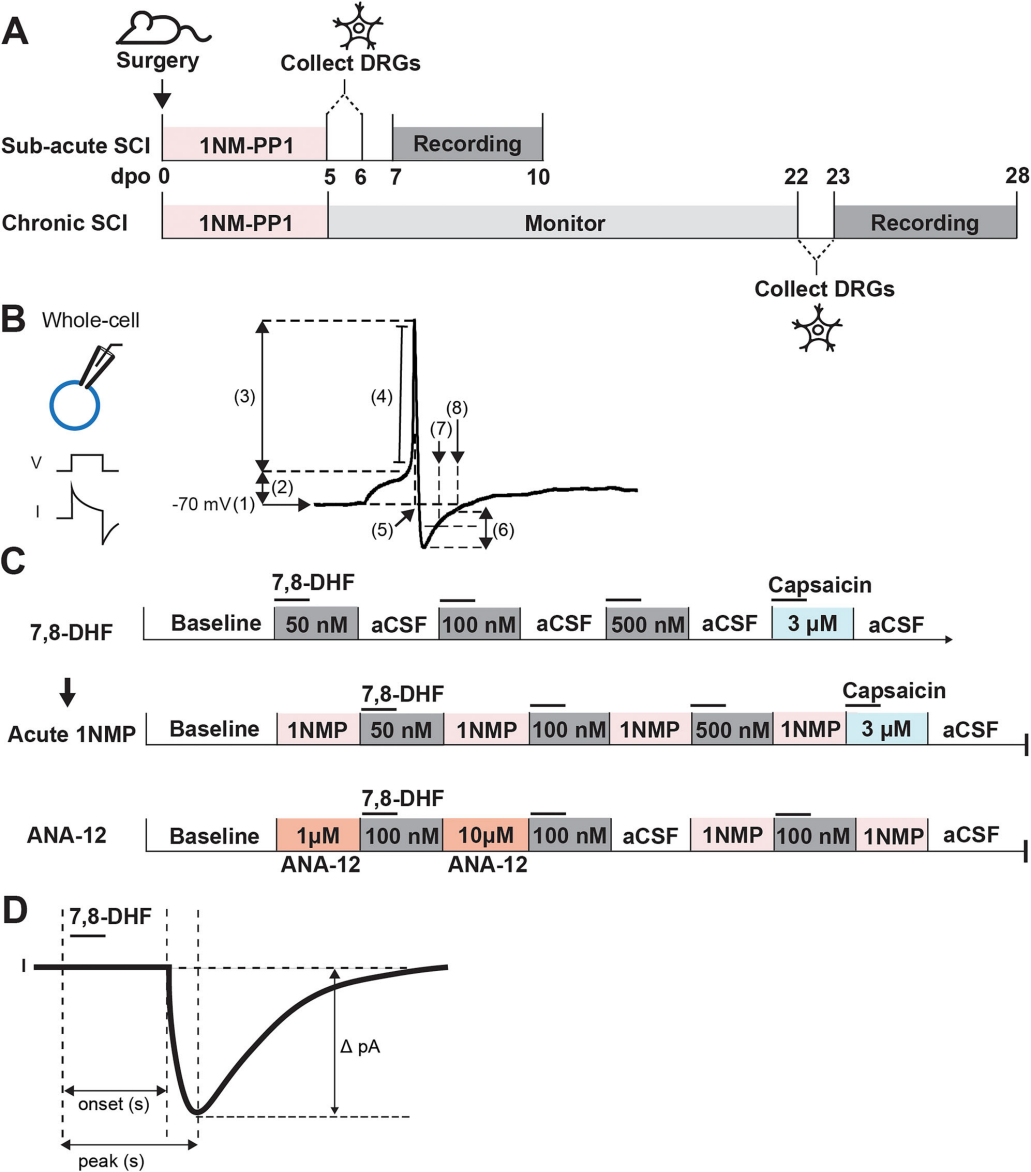
Illustration of the experimental design and assessment of electrophysiological properties. ***A***, Timeline of experimental procedures for both subacute and chronic SCI. For subacute SCI group, mice received 1NM-PP1 (1NMP) immediately following SCI surgery (0 d post operation, dpo) for 5 consecutive days. DRGs were collected on dpo 6. Patch-clamp recording began 24 h after dissociation up to 72 h. For chronic SCI group, 1NMP was also administered on dpo 0 for 5 d, but the animals were monitored for 3–4 weeks postinjury before DRG collection, dissociation, and electrophysiological recordings. ***B***, Left, Patch-clamp electrophysiology recording was used to assess neuronal properties in both voltage- and current-clamp configurations. Right, Key membrane properties were measured: (1) resting membrane potential (RMP, mV), (2) action potential (AP) threshold (mV), (3) AP amplitude (mV), (4) AP rise slope (mV/ms), (5) AP half-width (ms), (6) afterhyperpolarization (AHP) amplitude (mV), (7) AHP duration at 50% recovery (AHP_50_, ms), and (8) AHP duration at 80% recovery (AHP_80_, ms). ***C***, Top, Baseline activity was recorded during superfusion of artificial cerebrospinal fluid (aCSF), before sequential application of increasing concentrations of 7,8-DHF (50, 100, 500 nM), with aCSF washes between each concentration. Capsaicin (3 µM) was applied at the end of each recording to confirm that the recorded neuron is likely a nociceptor. Middle, For a subset of cells (*n* = 40), the protocol was repeated with the addition of 1 µM 1NMP to assess the effects of acute TrkB blockade in vitro. Bottom, Some cells were treated with two concentrations (1 and 10 µM) of ANA-12, a selective TrkB inhibitor, administered directly into the bath. ***D***, Quantification of inward currents elicited by 7,8-DHF treatment was done by measuring the largest difference in amplitude between the baseline and the current response (peak magnitude; Δ pA). Latency to respond and to peak response (s) were analyzed from the time the drug was added, to when the response begins and to the peak of the response, respectively.

Experiments were performed in adult male (*n* = 24) and female (*n* = 31) Ntrk2^F616A^ (F616) mice that enable selective, yet reversible pharmacogenetic inhibition of TrkB signaling (JAX # 022363; X. Chen et al., 2005). They were housed in standard cages in a vivarium on a 12 h light/dark cycle. Animals were fed standard rodent diets *ad libitum*. Experimental procedures were approved by the Animal Care and Use Committee of Emory University and conformed to national standards for the care and use of experimental animals and the American Physiological Society's “Guiding Principles in the Care and Use of Animals.” At the time of drug treatment and surgery, all animals were ∼8–12 weeks of age and weighed 22–26 g (males) and 16–22 g (females). An experimental timeline illustrating when the procedures described below were undertaken is provided in Figure 1*A*.

### Drug administration and pharmacological verification

F616 mice were treated with 1-(1,1-dimethylethyl)-3-(1-naphthalenylmethyl)-1H-pyrazolo[3,4-d]pyrimidin-4-amine (1NM-PP1 or 1NMP; #13330, Cayman Chemical), a small molecule, cell-permeable kinase inhibitor to block TrkB signaling systemically and reversibly, such as seen in previous studies (Mantilla et al., 2014; Martin et al., 2022). Consistent with the study by Martin et al. (2022), 1NMP was administered in drinking water at a final concentration of 5 μM (dissolved in dimethyl sulfoxide, DMSO). This treatment paradigm delayed the development of mechanical pain, and electrophysiological and pharmacological evaluation in acutely dissociated DRG neurons obtained from 1NMP- and Veh-treated F616 mice showed effectiveness of 1NMP inhibition. Therefore, this approach ensures consistency in examining cellular mechanisms underlying the observed behavioral attenuation. A cohort of mice were treated with an equal volume of DMSO in drinking water (dilution 1:10,000) and served as vehicle (Veh)-treated control. Mice were provided with 1NMP or Veh-treated water for 5 d, consuming ∼30–40 ml, comparable with previous reports.

### Surgical procedure

Mice were deeply anesthetized with isoflurane (5% gas; lowered to 2–3% once stable anesthesia was achieved). A skin incision, followed by a dorsal laminectomy, was performed to remove the vertebra over the spinal cord at T9–10. The exposed spinal cord was impacted at T10 under sterile conditions. Mice received midline contusion injuries with an Infinite Horizon Impactor (Precision Systems and Instrumentation) with 70 kdyne, zero dwell time impact onto the dorsal surface of the spinal cord, also as previously described (Parvin et al., 2021; Martin et al., 2022; Noble et al., 2022). After the impact, bilateral bruising of the dorsal spinal cord was carefully verified by examination under a dissecting microscope. The muscle and skin were sutured, and the wound area was treated with a topical triple antibiotic ointment (bacitracin-neomycin-polymyxin B). All mice recovered on a heated pad and were given meloxicam (5 mg/kg, subcutaneously), Baytril (2.5 mg/kg), and saline solution (0.5 ml) intraperitoneally, immediately after surgery for acute pain, infection, and hydration management. Baytril was subsequently given every morning for up to 7 d post operation (dpo) to minimize the risk of urinary tract or bladder infection in SCI animals. Mice bladders were manually expressed twice daily for the duration of experiments. Mice were assessed for impairment of locomotor function at 1 dpo using the Basso Mouse Scale (BMS; Basso et al., 2006) to ensure the effectiveness of the injury. Only SCI mice scoring 0 or 1 at 1 dpo were included in the study. Uninjured (Uninj in figures) mice, which did not receive laminectomy or contusion, nor subjected to any post-surgical manipulations, served as control. Uninjured and SCI F616 mice were treated with 1NMP or Veh in drinking water as described above. Experiments consisted of the following groups: Uninjured-Veh, Uninjured-1NMP, SCI-Veh, and SCI-1NMP. For SCI mice, 1NMP or Veh treatment began on the day of surgery (0 dpo).

### Dissociation of DRG neurons

Mice were deeply anesthetized and killed with isoflurane. DRGs were extracted from neural segments T4–lumbar (L) 2 and immediately placed in cold HBSS (#21-022-CV, Corning) grouped by the following segments: T4–T7, T8–T12, and T13–L2. Electrophysiological recordings were primarily made from the T8–T12 cells, which is the region near the site of the injury; however, the separation by region allows for the potential analysis of rostrocaudal differences. The DRGs were then incubated for enzymatic digestion in solution containing Dispase II (2.5 μ/ml; #04942078001, Sigma-Aldrich) and collagenase (200 μ/ml; #LS004176, Worthington Biochemical) in a 37°C water bath for 60 min and were gently inverted 4–5 times once every 15 min. Cells were dissociated 20–30 times in neurobasal medium-A (NB-A; #12349-015, Thermo Fisher Scientific) with 2% B-27 (#17504-044, Thermo Fisher Scientific), 1% penicillin/streptomycin (#17-602E, Lonza BioWhittaker), and 1% GlutaMAX (#35050061, Thermo Fisher Scientific) by trituration through a set of fire-polished glass pipettes in decreasing diameter. Dissociated cells were then centrifuged at 100 rpm for 3 min, after which the cells were resuspended and seeded at low density on coverslips coated with laminin (1 mg/ml; #12634-010, Sigma-Aldrich) and poly-ʟ-lysine (0.1 mg/ml; # p1274-100 mg, Sigma-Aldrich). Once seeded on 22 × 22 mm cover glass (#10026-140, VWR), the cells were incubated for 30 min. Two milliliter of NB-A medium were added per well for further incubation (12 ml total). Plates were kept in a 37°C incubator with 5% CO_2_ for at least 24 h before electrophysiological recording.

### Whole-cell recording from dissociated DRG neurons

An inverted microscope (Nikon Eclipse Ti-U) was used to identify small (15–30 μm) DRG neurons. Whole-cell patch recordings were made from such cells at room temperature using the conventional patch-clamp configuration. Signals were acquired with MultiClamp 700B amplifier (Molecular Devices), digitized at 10 kHz (Digidata 1440 A; Molecular Devices), and filtered at 1 kHz (Clampex 10.2, Molecular Devices). Patch electrodes with a resistance of 6–8 MΩ were pulled from borosilicate micropipettes (World Precision Instruments) using a Flaming/Brown P-97 micropipette puller (Sutter Instrument) and filled with solution containing (in mM) 140 K-gluconate, 11 EGTA, 10 HEPES, 1 CaCl_2_, 4 Mg-ATP, and 1 Na-GTP (pH 7.4 adjusted with KOH, osmolarity ranged from 290 to 300 mOsM). Artificial cerebrospinal fluid (aCSF) containing (in mM) 140 NaCl, 3 KCl, 2 MgCl_2_, 1.8 CaCl_2_, 10 glucose, 10 HEPES (pH 7.3 adjusted with KOH, ∼310–320 mOsM) was oxygenated with 95% O_2_–5% CO_2_ and continuously delivered to the recording chamber. Gentle negative pressure was applied to form a tight seal (resistance >1 GΩ) between the electrode tip and the cell membrane. The cell membrane was ruptured by applying gentle pressure to achieve the whole-cell configuration.

**Figure 2.**
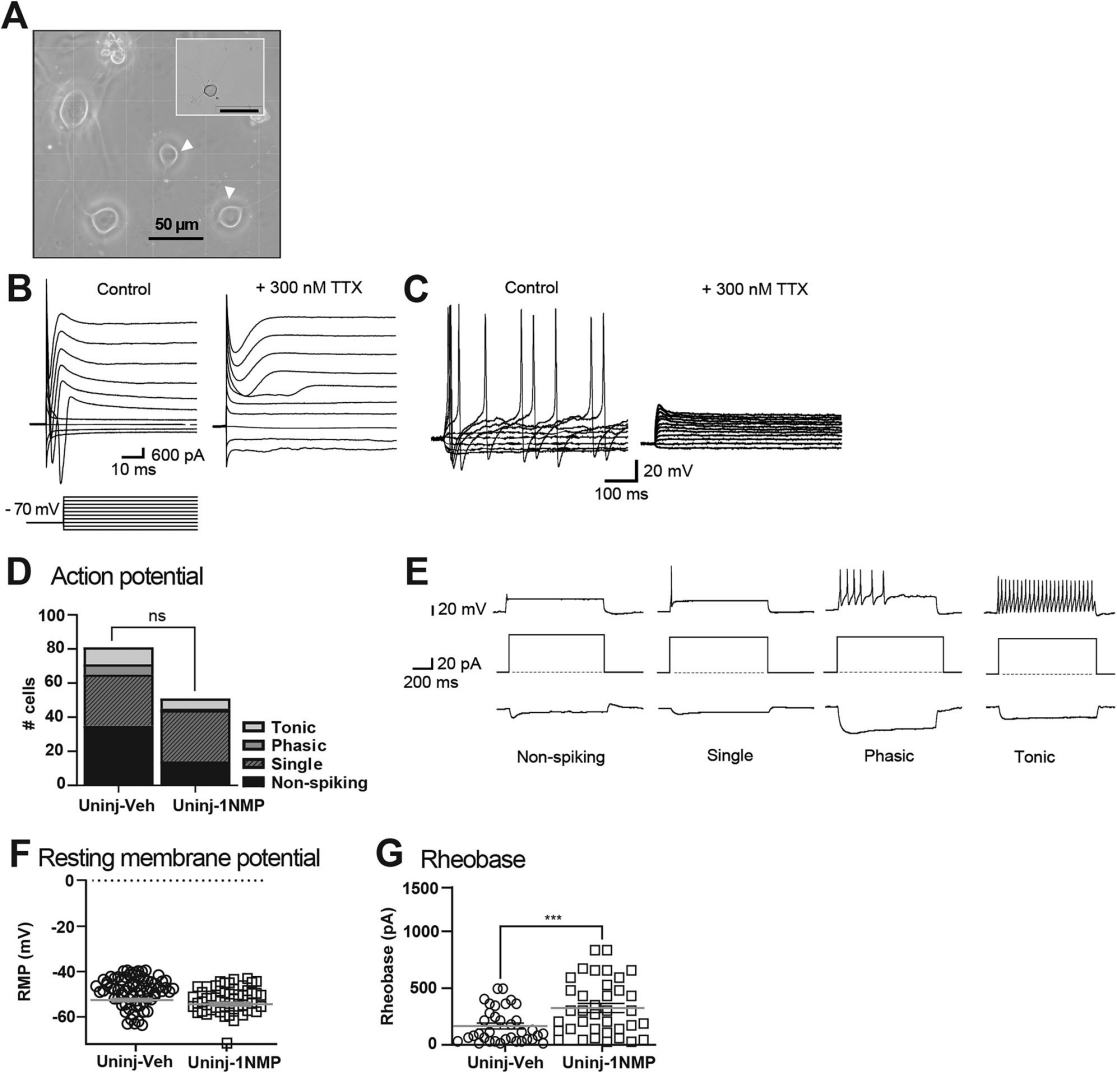
Characterization of dissociated DRG neurons. ***A***, Representative bright-field image (40×) showing the morphology of dissociated DRG neurons 24–72 h after plating and incubation at 37°C. Arrowheads indicate neurons typically selected to patch, in comparison with larger neurons. The inset image shows another cell in the same culture at lower magnification (10×). Scale bar, 50 µm. ***B***, Representative voltage-clamp recording of large voltage-gated Na^+^-mediated inward currents followed by K^+^-mediated outward currents in response to 20 ms hyperpolarizing and depolarizing steps. Neurons were held at −70 mV. In the right panel, the addition of 300 nM tetrodotoxin (TTX) blocked sodium-mediated inward currents. ***C***, Representative current-clamp recording showing multiple APs fired in a single neuron in response to a 20 ms depolarizing current injection through the recording electrode. Membrane time constant (***τ***) was determined using single exponential fitted to the first hyperpolarizing step, and input resistance (MΩ) was determined from the second hyperpolarizing step. The right panel shows the effects of TTX, which eliminated the APs. ***D***, Chi-square analysis of proportion of neurons categorized as nonspiking (veh, *n* = 35; 1NMP, *n* = 14), and single- (veh, *n* = 30; 1NMP, *n* = 30), phasic- (veh, *n* = 6; 1NMP, *n* = 1), and tonic- (veh, *n* = 10; 1NMP, *n* = 6) firing cells from Veh- and 1NMP-treated uninjured (Uninj) mice (*X*^2 ^= 7.121; df = 3; *p* = 0.0681). ***E***, Examples of nonspiking, single, phasic, and tonic firing in response to −60 mV depolarizing current injection, with corresponding hyperpolarizing steps depicted underneath. ***F***, Comparison of resting membrane potential (RMP) and ***G***, rheobase between Veh- and 1NMP-treated uninjured populations. Responses of individual neurons (circles, Veh; squares, 1NMP) and sample means (horizontal lines) are both shown. The neuronal RMP did not change after treatment with 1NMP (*p* = 0.3120). The rheobase, defined as the minimum amount of depolarizing current needed to evoke a single AP, significantly increased with 1NMP treatment (***p < 0.001).

Once in whole-cell configuration, the Clampex Membrane Test program (Molecular Devices) was used to determine *C_m_* and membrane resistance *R_m_* from a holding potential of −70 mV in voltage-clamp configuration. While held at −70 mV in voltage-clamp mode, hyperpolarizing (−40 mV) and a series of depolarizing (Δ20 mV) current steps were undertaken to identify inward current response and to obtain estimates of several membrane properties. The voltage-gated sodium channel (VGSC) blocker, tetrodotoxin (TTX; 300 nM), was bath applied to a subset of neurons (*n* = 10). Inward current in both voltage- and current-clamp configuration was blocked by TTX (Fig. 2*B*,*C*). The configuration was then switched to bridge mode (*I* = 0) and the resting membrane potential (RMP) was recorded. Other electrophysiological properties to determine the excitability of the neurons were recorded using current-clamp configuration, at −60 mV, with ascending series of 2 ms long depolarizing pulses until action potential (AP) was evoked. To observe spontaneous firing, neurons were recorded in current-clamp gap-free mode over one 1 min period at their RMP.

### Assessment of electrophysiological properties

To compare the changes in neuronal activity before and after drug application or injury, various membrane properties were measured (depicted in Fig. 1*B*). RMP was noted after a stable patch was established and was carefully monitored for the duration of the experiment. Rheobase, the minimum current required to evoke an AP, and the AP voltage threshold, which measures the voltage at the onset of the AP, were calculated at rest and after drug treatment. The AP and afterhyperpolarization (AHP) were determined from the first trace with an AP spike. AP amplitude was measured between the AP threshold and AP peak. The AHP amplitude was measured from the peak of the hyperpolarization to the baseline. The AHP duration can be measured in several ways. Here, AHP duration, as milliseconds, was measured at 50% (AHP_50_) and 80% (AHP_80_) recovery of the AHP back to baseline, as previously described (Koerber et al., 1988; Lawson et al., 1996). The instantaneous firing frequency describes the rate of AP spike generation over the duration of the current input (2 ms) and was obtained from the first depolarizing step with more than one AP spike. The input resistance (*R*_in_) for each cell was obtained from the slope of the second trace in response to a series of hyperpolarizing currents, and the membrane time constant, tau (*τ*), was determined with a single exponential fitted to the hyperpolarizing step. Cell capacitance was recorded after the whole-cell configuration and back calculated from *R*_in_ and tau using *R*_in_*C*_in _= *τ*.

### Preparation and application of drugs

All drugs were delivered by bath perfusion during gap-free voltage-clamp recording at −60 mV as illustrated in Figure 1*C*. Drugs were first prepared as concentrated stock solution in aCSF and stored at −20°C. Stock solutions of 7,8-DHF (#D1916, Tokyo Chemical Industry) and capsaicin (#211275, EMD Millipore) were dissolved in 100% DMSO. Drug stock solutions were diluted in aCSF on the day of recording. The final concentration of DMSO was minimal and not expected to influence neuronal activity (Galvao et al., 2014; Zhang et al., 2017). 7,8-DHF was diluted to final concentrations of 50, 100, and 500 nM, which were applied 1–2 min after the start of the recording before being washed out for at least 2 min.

For acute 1NMP application, the neurons were recorded to establish a baseline for response to 7,8-DHF in an identical protocol. After establishing the baseline response to 7,8-DHF, 1 μM 1NMP was superfused, followed by reapplication of the same concentrations (50, 100, 500 nM) of 7,8-DHF to observe the acute effects of 1NMP on TrkB-mediated currents. Some cells were also tested with a TrkB-specific inhibitor, ANA-12 (Cazorla et al., 2011). This experiment was undertaken to further confirm that 7,8-DHF-induced inward current is indeed mediated by engaging TrkB. ANA-12 was first dissolved in DMSO at a concentration of 20 mM. Following a baseline response to 7,8-DHF, ANA-12 (1 and 10 μM) was bath applied in the presence of 100 nM 7,8-DHF.

Capsaicin sensitivity is widely considered a nociceptive marker because many nociceptors express the Transient receptor potential vanilloid 1 (TRPV1; Cardenas et al., 1995; Caterina et al., 1997; Petruska et al., 2000). Here, capsaicin sensitivity was tested in all recorded neurons. In a subset of sensory neurons (*n* = 96), response to capsaicin was first assessed by delivering increasing concentrations of capsaicin (0.3, 1, 3, 10, 30 μM; final concentrations in aCSF diluted from a 3 mM stock solution in DMSO). Consistent with other studies, 3 μM was determined to produce the most reliable response (data not shown). Therefore, in all neurons, after 7,8-DHF was completely washed out by bathing with aCSF for 10 min, 3 μM capsaicin was bath applied for 1 min, followed by another aCSF wash.

### Western blot for TrkB expression

At 7 dpo, nine mice (five SCI and four uninjured) were deeply anesthetized with isoflurane and oxygen (as described above), and T4 to L2 DRGs were rapidly removed and flash frozen in liquid nitrogen. Total protein was extracted using RIPA lysis buffer and quantified using the bicinchoninic acid protein assay. Protein samples were then diluted in the Laemmli sample buffer and stored at −80°C for Western blot. Equal amounts of total protein (30 μg) were assayed through SDS-PAGE using 12% Tris-HCl gels and then transferred to polyvinylidene difluoride membranes (#03010040001, Millipore). Blots were blocked for 1 h in 5% blotting grade milk (Bio-Rad Laboratories) in Tris-buffered saline Tween-20 (TBST). The blots were incubated overnight at 4°C in primary antibodies: TrkB (1:1,000; #AF1494, RRID:AB_2155264; R&D Systems) generated in goat and β-tubulin (1:1,000; #05-661, RRID:AB_309885; Upstate Cell Signaling) generated in mouse. The following day, blots were washed in TBST and then incubated in HRP-conjugated donkey anti-goat (1:10,000; #PA1-28805, RRID:AB_10988865; Thermo Fisher Scientific) or goat anti-mouse secondary antibodies (1:2,500; #31430, RRID:AB_228307; Thermo Fisher Scientific). The blots were developed with standard enhanced chemiluminescence and imaged with Azure Biosystems c600 Western blot Imaging System. Ratios of the integrated densitometry of each protein of interest to the loading control (β-tubulin) were calculated with AlphaView Software (ProteinSimple), normalized to uninjured controls.

### Immunocytochemistry

Coverslips containing dissociated DRG neurons were fixed in 4% PFA at room temperature and then washed briefly in PBS. After washing, culture dishes were permeabilized in 0.1% Triton X-100 and then blocked in 10% Normal Goat Serum in PBS-Tween 20 at room temperature. Samples were then incubated with primary antibody (diluted in the blocking solution) overnight at 4°C. The following primary antibodies were used: mouse anti-NeuN (1:400; #MAB377, RRID:AB_2298772; EMD Millipore), rabbit anti-TrkB (1:2,000; #AF1494, RRID:AB_2155264; R&D Systems). Following three 5 min washes in PBS, secondary antibodies were applied for 1 h at room temperature: Alexa Fluor 488 (1:100; #A-11008, RRID:AB_143165; Invitrogen), 546 (1:250; #A-11035, RRID:AB_2534093; Invitrogen). Samples were mounted onto glass slides with ProLong Gold antifade reagent (#P36934, Invitrogen) followed by image capture under Keyence microscope using Keyence BZ-X710 imaging software.

### Statistical analysis

Mice were randomly assigned to each experimental group. All statistical measures and analyses were undertaken with GraphPad Prism 10 (GraphPad Software). While experimenters could not be blinded to SCI versus uninjured groups, experimenters performing behavioral tests and subsequent statistical analyses were blinded to treatment (1NMP vs Veh). All statistical data in text and figures are presented as mean ± standard error of the mean (SEM) along with the number of samples analyzed (*n*). Comparison between groups was accomplished using two-way repeated-measures (RM) ANOVA as injury condition/drug treatment as the between-subjects factor. For categorical comparisons, chi-square (*χ*^2^) test or Fisher's exact test was used. Student's *t* test were used where only two groups were being compared, and *p *< 0.05 was considered statistically significant. Cells were excluded if they did not exhibit an inward current in response to depolarizing steps or if their RMP was more depolarized than −40 mV. Outliers were determined using Grubbs’ test.

## Results

Forty-six F616 mice (18 males and 28 females) were used for electrophysiology as follows: Uninjured Veh (15), Uninjured-1NMP (8), SCI-Veh (12), and SCI-1NMP (11). An additional nine mice were used for Western blot (four uninjured and five SCI). The specific breakdown of animals used in the current study is summarized in Table 1. The contusion at T10 produced hindlimb paralysis in all mice in the study and was confirmed by BMS score (SCI group mean BMS score, 0.1; 1 d after injury). F616 mice express a mutated TrkB that enables selective inhibition of TrkB signaling with the small molecule kinase inhibitor, 1NMP. Mice were treated with 1NMP (or Veh) in drinking water to inhibit TrkB signaling systemically to evaluate contribution of TrkB to neural mechanisms of pain hypersensitivity after SCI.

**Table 1.** Summary of animals used

Condition	Uninjured						SCI					
Experiments	Electrophysiology				Immunoblotting		Electrophysiology				Immunoblotting	
Treatment	Veh		1NMP				Veh		1NMP			
Sex	Males	Females	Males	Females	Males	Females	Males	Females	Males	Females	Males	Females
*n* animals	3	12	4	4	3	1	2	10	9	2	3	2
	23				4		23				5	
Total per group	27						28					

Table summarizing total number of animals used in electrophysiological and immunoblotting experiments in each injury and treatment conditions. Veh, vehicle-treated; 1NMP, animals that were treated with 1NMP in drinking water.

### Morphologies and properties of DRG neurons from uninjured and SCI F616 mice

T4 to L2 DRGs were collected from animals in each group to be dissociated and cultured for 24 h before recording. Dissociated neurons displayed a smooth, clear membrane surface and sometimes other smaller cells attached, possibly satellite glial cells. Most neurons developed neurites with more complex branching the longer they were incubated (Fig. 2*A*). Visually, there were no apparent differences in cultured DRG neurons from Uninjured-Veh mice to cultured neurons from other experimental groups (Uninjured-1NMP, SCI-Veh, and SCI-1NMP).

Neurons with small diameters (typically <30 µm) were selected for whole-cell patch-clamp recording as previously described (Davidson et al., 2014). Once a stable recording was established, RMP was determined. Only the neurons that displayed an TTX-sensitive inward current in response to the depolarizing steps (Fig. 2*B*) and had a RMP more negative than or −40 mV (less than −40 mV; Fig. 2*F*) were evaluated (total *n* = 124). Recording was undertaken in both voltage-clamp and current-clamp configurations (Fig. 2*B*,*C*) for subsequent analyses of various firing and membrane properties (Fig. 1*B*, right).

The cells displayed a range of firing properties in response to 20 ms pulses of depolarizing steps ranging from 20 to 120 pA that could be classified into four categories. Most of the neurons fired one AP spike (single), which was followed by AHP, both of which are characteristic of typical DRG neurons (Peacock et al., 1973). Some neurons exhibited an early train of APs that diminished (phasic), and others fired tonically, exhibiting sustained multiple AP firing pattern throughout the whole depolarizing step once threshold was reached (tonic). These multiple firing patterns are comparable with firing observed in dorsal horn neurons (Hochman et al., 1997). Interestingly, some cells did not fire an AP in response to depolarizing current steps (nonspiking neurons). Notably, these nonspiking cells were included in the neurons that were treated with TTX and were confirmed to be neurons by the presence of voltage-gated sodium currents. These neurons were also viable, as evidenced by their response to drug treatments. Representative traces of each category are shown in Figure 2*E*. In 81 DRG cells from Veh-treated mice, most neurons fired a single spike (*n* = 30) or did not fire an AP (*n* = 35). Fewer neurons fired phasic (*n* = 6) or tonic spikes (*n* = 10). Similarly, in neurons obtained from 1NMP-treated uninjured mice, 30 of 51 neurons fired once and 14 did not fire an AP, while fewer neurons fired few (*n* = 1) or tonic (*n* = 6) APs. A chi-square test showed no statistical difference in the distribution of AP firing between the two treatment groups (*X*^2 ^= 7.121; df = 3; *p* = 0.0681; Fig. 2*D*). This result indicates that 1NMP is not altering the ability of cells to reach the threshold for firing APs, and once the threshold is reached, 1NMP does not alter their firing patterns, including the likelihood of neurons to fire repetitively. Indeed, 1NMP treatment did not change basal RMP of the DRG neurons (Veh −50.0 ± 0.8; 1NMP −51.3 ± 0.9; Fig. 2*F*); however, it significantly increased the rheobase (Veh 203.1 ± 30.6; 1NMP 409.2 ± 48.9; *p* = 0.0007; Fig. 2*G*), corresponding to the reduction in number of cells that were able to fire, repetitively.

### TrkB agonist induces inward currents in small-diameter DRG neurons

**Figure 3.**
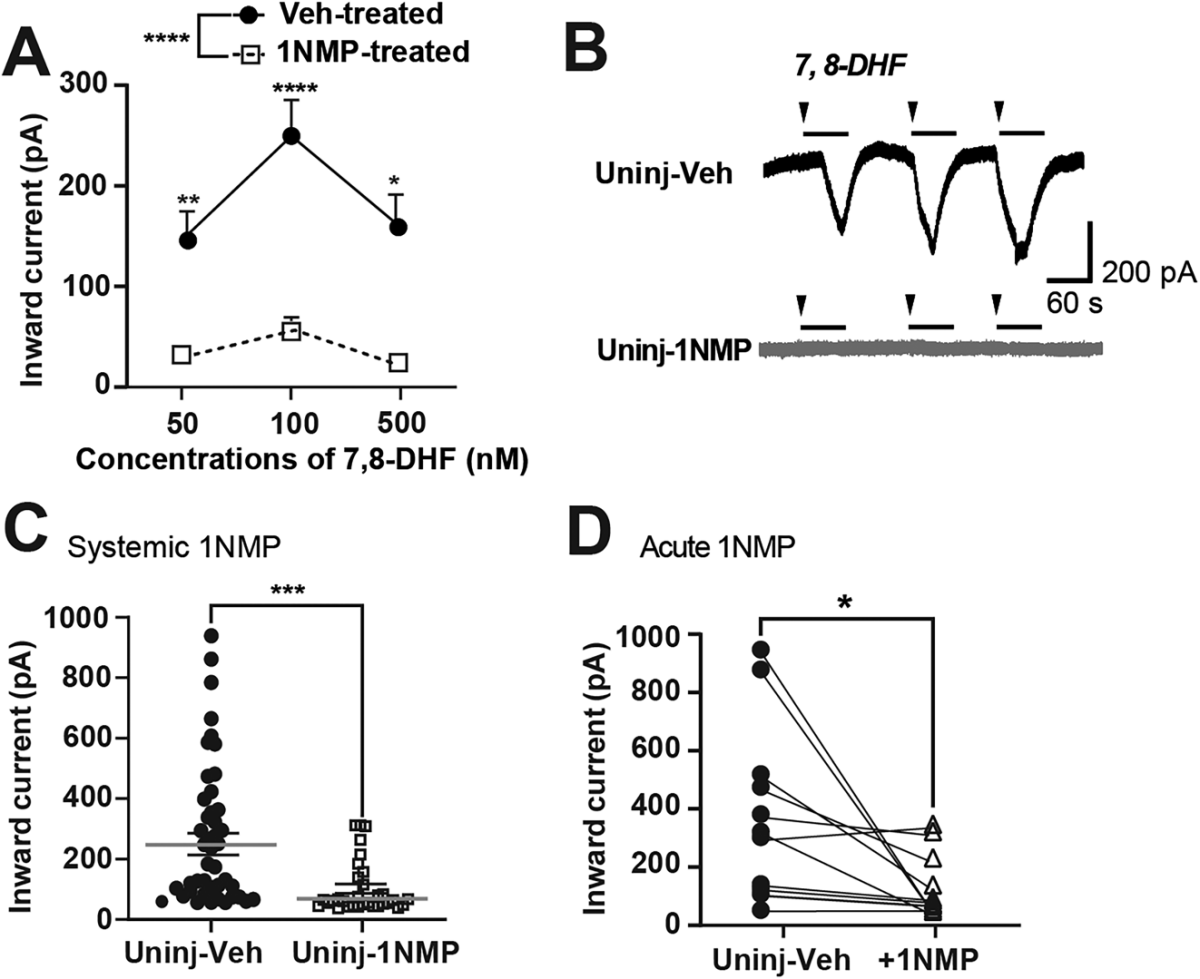
7,8 DHF-induced inward current in DRG neurons. ***A***, Analysis of the peak magnitude of the inward current elicited by increasing concentrations of 7,8-DHF (50, 100, or 500 nM) in DRG neurons from Uninjured-Veh (filled circle, solid line) and Uninjured-1NMP (open squares, dotted line) mice revealed a significant effect of treatment (*F*_(5,162) _= 11.6; *p* < 0.0001; one-way ANOVA). Post hoc comparisons showed significant differences between Veh- and 1NMP-treated groups at 50 nM (**p = 0.0042), 100 nM (****p < 0.0001), and 500 nM (*p = 0.0137). ***B***, Representative traces of whole-cell voltage-clamp recordings (holding potential −60 mV) demonstrate significant reduction in inward current in a neuron from 1NMP-treated animals (bottom) compared with a neuron from Uninjured-Veh animals (top). Arrowheads indicate the point of drug introduction to the bath, and the bar indicates the duration of 7,8-DHF perfusion into the bath (1 min). ***C***, Systemic 1NMP treatment (in drinking water) significantly reduced the inward current induced by 100 nM 7,8-DHF compared with current amplitudes in induced in neurons from uninjured animals (***p = 0.0001). ***D***, Paired comparison of inward currents in neurons from uninjured animals subjected to acute 1NMP treatment in vitro revealed a significantly reduced current amplitude during acute 1NMP application (*p < 0.05; paired *t* test). Data points represent individual neurons; horizontal lines indicate mean ± SEM. Lines between the two groups in ***D*** represent pair relationship belonging to the same cell.

TrkB activation in the DRG neurons was assessed using the small molecule TrkB agonist, 7,8-DHF (Liu et al., 2016) in voltage clamp at −60 mV. Following a period of stable baseline recording (∼2 min), 50, 100, and 500 nM of 7,8-DHF was bath applied to DRG neurons for ∼1 min with washout periods of at least 2 min in between, during which the response returned to baseline. In a total of 95 neurons, 7,8-DHF-induced robust inward current response at all concentrations (50 nM, 151.0 ± 23.7 pA, *n* = 30; 100 nM, 249.6 ± 35.4 pA, *n* = 45; 500 nM, 163.0 ± 28.4 pA, *n* = 20; Fig. 3*A*,*B*). The inward current peak amplitudes were compared with those evoked in neurons obtained from mice that were orally administered 1NMP (Uninjured-1NMP) for systemic inhibition of TrkB. One-way ANOVA revealed that systemic 1NMP treatment (Uninjured-1NMP) significantly reduced 7,8-DHF-induced current in DRG neurons (*F*_(5,162) _= 11.6; *p* < 0.0001; Fig. 3*A*). In fact, 1NMP reduced the current at all three concentrations (50 nM, 32.4 ± 4.3 pA, *n* = 28; 100 nM, 56.5 ± 12.8 pA, *n* = 37; 500 nM 22.9 ± 3.2 pA, *n* = 20, open squares) compared with the corresponding Veh-treated group (Fig. 3*A–C*, filled circles). Of the three concentrations of 7,8-DHF, the response evoked with 100 nM 7,8-DHF was the most robust. Thus, the remaining electrophysiological assessments, including results shown in Figure 3*C*,*D*, were performed with 100 nM 7,8-DHF as the primary concentration.

We assessed the effect of bath-applied 1 µM 1NMP (dissolved in aCSF from a 1 mM stock 1NMP) on 7,8-DHF-evoked responses in a subset of neurons (*n* = 31) obtained from Veh-treated uninjured mice (acute-1NMP). Acute 1NMP did not reduce the amplitude of the evoked 7,8-DHF current at all three concentrations, when compared with the Veh-treated population (*F*_(5,119) _= 1.6; *p* = 0.179). However, because concentration–response evaluation was not conducted for acute 1NMP application, 1 µM may be insufficient for effective inhibition. Despite this observation, in 10 cells, we tested the effect of 100 nM 7,8 DHF before and during 1NMP and evaluated their responses using paired analysis. The results revealed that acute 1NMP application significantly reduced the 7,8-DHF-induced inward current from 342.0 ± 104.3 pA (before 1NMP) to 65.32 ± 17.32 pA (during 1NMP; *p* = 0.0256; Fig. 3*D*), confirming that 1NMP treatment attenuates TrkB signaling in the F616 mice. To further confirm that the agonist-induced inward currents observed in sensory neurons were mediated by TrkB, a TrkB-specific inhibitor, ANA-12 (Cazorla et al., 2011), was introduced to five cells as a secondary method of TrkB inhibition. Concentration-dependent reductions in 100 nM 7,8-DHF-evoked inward currents were observed with 1 and 10 μM ANA-12 (data not shown), with a magnitude of response that was qualitatively comparable with that seen with acutely applied 1NMP. Specifically, 10 μM ANA-12 decreased 7,8-DHF currents from 325.1 ± 99.6 to 119.7 ± 60.6.3 pA (63% decrease in amplitude). These results demonstrate that 7,8-DHF is inducing neuronal response through TrkB activation.

### Decreased TrkB-mediated inward currents in the DRGs following SCI

**Figure 4.**
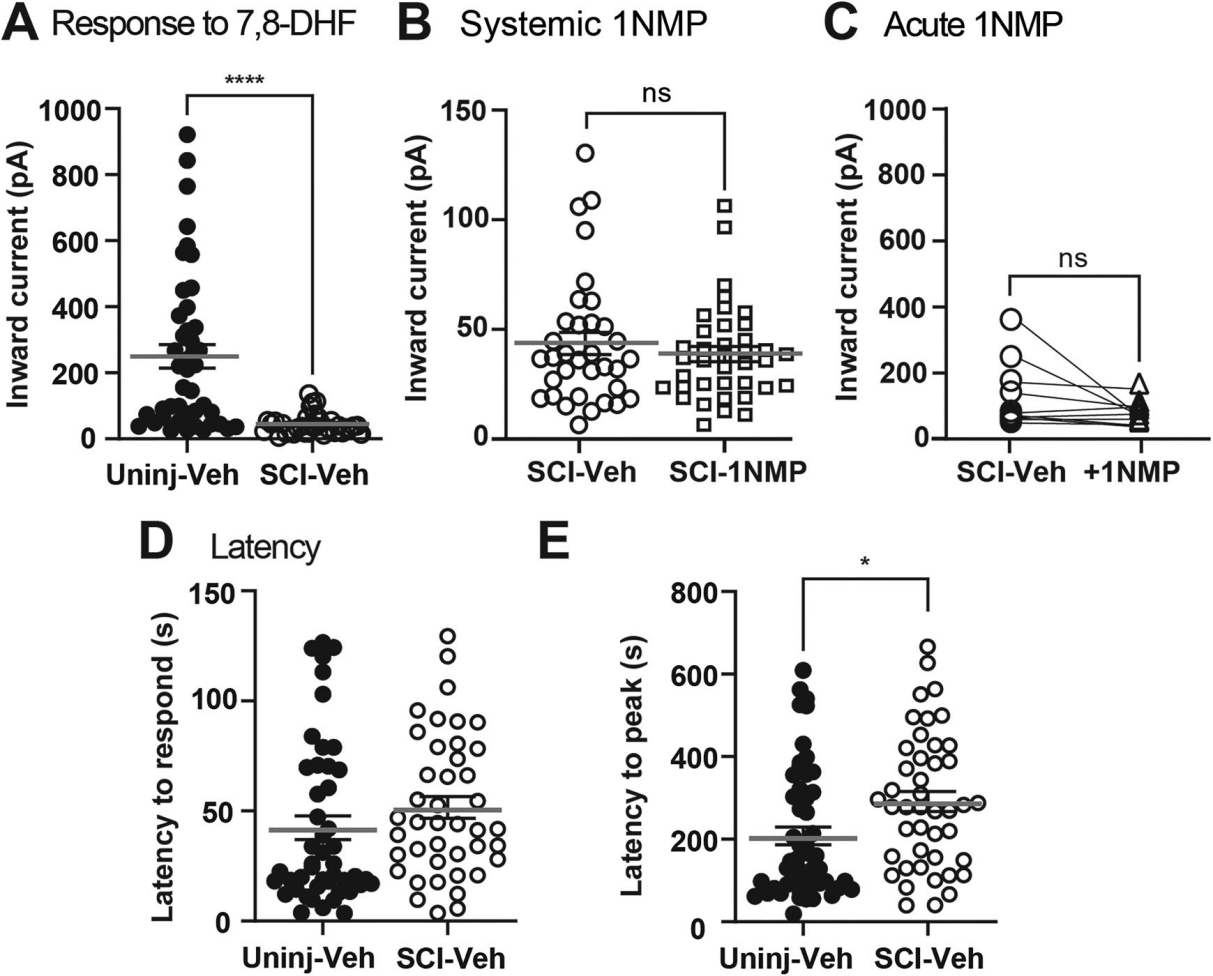
SCI decreased 7,8-DHF-induced inward current in DRG neurons. ***A***, Plot of the inward current produced by 100 nM 7,8-DHF in neurons obtained from Veh-treated Uninjured and SCI mice shows SCI significantly reduced 7,8-DHF-induced inward current amplitude (****p < 0.0001). ***B***, Compared with DRG neurons from SCI-Veh animals, systemic 1NMP treatment in SCI subjects had no additional effect on 7,8-DHF-evoked inward currents (*p* = 0.1183). ***C***, Paired comparison of inward currents in neurons from SCI-Veh animals subjected to acute 1NMP treatment in vitro showed no difference in 7,8 DHF-evoked current (*n* = 10). ***D***, ***E***, Latency to onset of 7,8-DHF-induced current remained unchanged (*p* = 0.2163), while latency to peak current (peak magnitude) was significantly increased after SCI (*p = 0.0117). Data points represent individual neurons; horizontal lines indicate mean ± SEM. Lines between the two groups in ***C*** represent pair relationship belonging to the same cell.

The next step in this study was to assess 7,8-DHF-evoked responses in dissociated sensory neurons after SCI. As described above, mice received a T10 contusion injury and were immediately treated with either 1NMP (SCI-1NMP) or vehicle (SCI-Veh) for 5 d (0–5 dpo). T4-L2 DRG neurons were recorded at subacute (5–7 dpo) or chronic (21–28 dpo) phases after SCI (Fig. 1*A*). Since no significant differences were found in the inward current amplitude between neurons from the subacute and chronic groups, the data were combined to increase statistical power and henceforth referred to as SCI. Surprisingly, compared with the inward currents generated in neurons from Uninjured-Veh mice (249.6 ± 35.9 pA), 100 nM 7,8-DHF evoked a significantly decreased inward current response in SCI-Veh populations (44.5 ± 33.7 pA; *p* = 0.0004; Fig. 4*A*). In neurons from SCI-1NMP mice, 7,8-DHF evoked an even smaller inward current (34.9 ± 3.2) that was not statistically different to the SCI-Veh group (*p* = 0.1183; Fig. 4*B*) but different to both uninjured groups. Additionally, 1 µM 1NMP was bath applied acutely to 10 neurons from SCI-Veh group after recording the 100 nM 7,8-DHF-induced inward current. In these neurons, acute 1NMP application caused a reduction in inward current (from 102 ± 31.5 to 50.8 ± 10.9 pA), although this decrease was not statistically significant in paired analysis (*p* = 0.1034; Fig. 4*C*). Unlike the robust reduction observed in the Uninjured group, the response to 1NMP in neurons from injured F616 mice was less pronounced. The lack of significant 1NMP-induced reduction in SCI neurons may reflect a floor effect in the inward current magnitude following SCI, rather than a lack of TrkB inhibition.

### SCI increases latency to peak of TrkB induced current

Inward current amplitude can be influenced by properties and dynamics of the channels mediating the current; thus, potential changes in TrkB channel activation after SCI was investigated by evaluating latency to respond and to peak amplitude of the 7,8-DHF current (Fig. 1*D*). Between the Uninjured-Veh (42.5 ± 5.4 s) and SCI-Veh neurons (51.6 ± 4.9 s), latency to respond to bath-applied 7,8-DHF did not change (*p* = 0.2163; Fig. 4*D*). However, SCI (291.5 ± 24.5 s) increased the latency to peak response compared with Uninjured-Veh (207.8 ± 21.6 s; *p* = 0.0117; Fig. 4*E*). 1NMP treatment significantly decreased latency to peak response after SCI (149.1 ± 13.8; *p* < 0.0001) but had no effect on the uninjured group (170.2 ± 19.03; *p* = 0.2100; data not shown).

### Small-diameter DRG neurons exhibit differential changes in excitability after SCI

**Figure 5.**
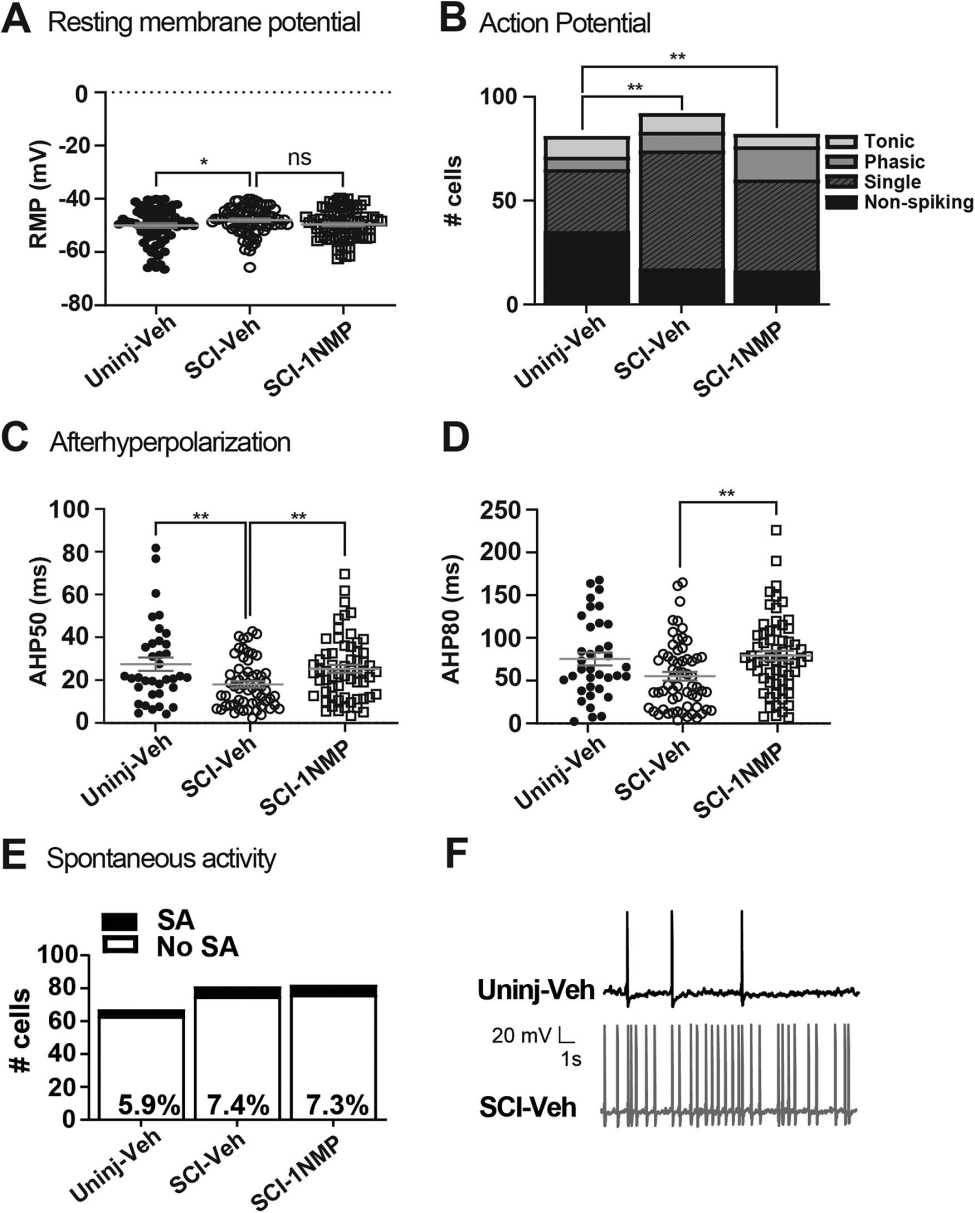
Changes in some neuronal properties after SCI. ***A***, Following SCI, RMP was significantly more depolarized (*p = 0.0391). ***B***, Chi-square analysis comparing the number of DRG neurons classified as nonspiking, single, phasic, and tonic AP firing revealed that SCI significantly increased the proportion of neurons that fire a single AP (*X*^2 ^= 14.62; df = 3; *p* = 0.0022). 1NMP treatment after SCI failed to return RMP or firing patterns to uninjured-vehicle measures. ***C***, SCI significantly reduced the AHP_50_ duration (*p = 0.0039) while ***D***, AHP_80_ was unchanged (*p* = 0.1023). 1NMP treatment after SCI significantly increased both AHP_50_ (18 ± 1.46 to 25.24 ± 1.86 ms; *p* = 0.0029) and AHP_80_ (54.64 ± 5.08 to 79.05 ± 5.71 ms; *p* = 0.0018) compared with SCI-Veh, and returning then to values not different to Uninjured-Veh. ***E***, Bar graphs represent number of cells/total cells recorded and examined for spontaneous activity (SA). Neither SCI nor 1NMP after SCI changed the proportion of DRG neurons displaying SA (7.41 and 7.3%, respectively) compared with neurons obtained from the uninjured vehicle group (5.9%). ***F***, Representative traces of spontaneous firing in current-clamp mode at −60 mV in neurons examined from uninjured (top) and SCI (bottom) groups. In ***A***, ***C***, and ***D***, data points represent individual neurons; horizontal lines indicate mean ± SEM.

Passive and active electrophysiological properties were analyzed to investigate potential mechanisms by which SCI or 1NMP alters TrkB-mediated neuronal excitability (Fig. 1*B*, right). Thus, electrical properties were monitored and noted after establishment of a stable patch and after each inward current recording in current-clamp configuration before and after acute treatments. Although some properties are highlighted in Figure 5, the analyzed results for all properties are summarized in Table 2. Changes in passive neuronal properties were examined, such as input resistance (*R*_in_), membrane time constant (***τ****_m_*), and membrane capacitance (*C_m_*), in addition to RMP. Measurements were obtained from an average of 287 cells across all four treatment and injury groups (some cells were excluded by the Grubbs’ test for outliers in addition to not meeting the predetermined criterion for the RMP). 1NMP treatment (−51.3 ± 0.9 mV) did not change the RMP (−50.0 ± 0.8 mV; *p* = 0.3120; Fig. 2*F*). Compared with uninjured-Veh-treated group (229.2 ± 21.5 MΩ; 95.5 ± 8.2 pF), 1NMP significantly decreased *R*_in_ (143.1 ± 14.6 MΩ; *p* = 0.0034) and increased *C_m_* (148.9 ± 16.3 pF; *p* = 0.0026) in uninjured cells (Table 2). Interestingly, 1NMP increased the rheobase in cells from Uninjured-1NMP animals (from 203.1 ± 30.6 to 409.2 ± 48.9 pA; *p* = 0.0007; Fig. 2*G*) but decreased it in neurons from SCI mice [261.9 ± 32.2 pA (SCI-Veh) compared with 154.4 ± 18.2 pA (SCI-1NMP); *p* = 0.0061; Table 2]. The threshold of AP generation, amplitudes, half-widths, and rise slopes of APs and firing frequencies between all groups were measured and compared as well (Fig. 1*B*, right). APs were evoked by 20 ms depolarizing current injections, and the first AP of the train was used for analysis.

**Table 2.** Summary of electrophysiological properties

Property	Uninj-Veh	Uninj-1NMP	SCI-Veh	SCI-1NMP	Uninj-Veh vs Uninj-1NMP		Uninj-Veh vs SCI-Veh		SCI-Veh vs SCI-1NMP	
RMP (mV)	−50.0 ± 0.8 (72)	−51.3 ± 0.9 (52)	−48.0 ± 0.6 (81)	−49.6 ± 0.6 (82)	NS		*	**0** **.** **039**	NS	
Rheobase (pA)	203.1 ± 30.6 (35)	409.2 ± 48.9 (37)	261.9 ± 32.2 (64)	154.4 ± 18.2 (56)	***	**0.0007**	NS		**	**0.0061**
*R*_in_ (MΩ)	229.2 ± 21.5 (62)	143.1 ± 14.6 (42)	253.5 ± 22.7 (70)	316.8 ± 26.4 (81)	**	**0.0034**	NS		NS	
Membrane time constant (ms)	27.1 ± 2.2 (68)	23.5 ± 2.4 (47)	25.6 ± 2.3 (87)	23.0 ± 1.7 (81)	NS		NS		NS	
Capacitance (pF)	95.5 ± 8.2 (57)	148.9 ± 16.3 (47)	79.9 ± 4.7 (74)	77.6 ± 4.8 (77)	**	**0.0026**	NS		NS	
AP threshold at −60 mV	34.1 ± 2.1 (38)	34.8 ± 2.2 (38)	34.6 ± 2.2 (66)	35.2 ± 1.5 (66)	NS		NS		NS	
AP amplitude (mV)	91.1 ± 4.0 (38)	86.7 ± 3.9 (41)	87.4 ± 2.3 (68)	94.3 ± 2.2 (66)	NS		NS		*	**0.0303**
AP half-width (ms)	4.6 ± 0.4 (42)	4.7 ± 0.5 (34)	4.9 ± 0.4 (66)	5.0 ± 0.3 (66)	NS		NS		NS	
AP rise slope (mV/ms)	3.1 ± 0.5 (37)	4.4 ± 0.6 (35)	3.1 ± 0.3 (60)	3.0 ± 0.3 (61)	NS		NS		NS	
Instantaneous Firing Frequency	6.1 ± 1.0 (14)	5.7 ± 1.5 (8)	7.4 ± 1.3 (16)	5.9 ± 0.8 (24)	NS		NS		NS	
AHP amplitude (mV)	17.7 ± 1.5 (38)	15.4 ± 1.0 (38)	15.7 ± 1.0 (66)	18.2 ± 1.0 (65)	NS		NS		NS	
AHP_50_ duration (ms)	27.4 ± 3.2 (36)	19.8.1 ± 1.8 (36)	18.0 ± 1.5 (59)	25.2 ± 1.9 (62)	*	**0.0404**	**	**0.0030**	**	**0.0029**
AHP_80_ duration (ms)	72.1 ± 7.6 (36)	68.1 ± 8.6 (37)	54.6 ± 5.1 (61)	79.1 ± 5.8 (62)	NS		NS		**	**0.0018**

Electrophysiological properties of small dissociated DRG neurons. RMP, resting membrane potential; AP, action potential; AHP, afterhyperpolarization; *R*_in_, input resistance. Numbers shown are mean ± SEM, with the number of neurons (*n*) indicated in parentheses. Data were collected from DRG neurons from both male and female animals across all experimental groups. Statistical comparisons were made using Student’s *t*-test. (*p < 0.05; **p < 0.01; ***p < 0.001; NS [not significant]). Statistically significant changes are also indicated in bold.

Comparative analyses were also undertaken in neurons obtained from SCI mice. Notably, SCI led to a less negative RMP (from −50.0 ± 0.8 mV to −48.0 ± 0.6 mV; *p* = 0.0391; Fig. 5*A*), and although rheobase also trended upward, it was not statistically significant (*p* = 0.2351; Table 2). In a total of 92 neurons from SCI-Veh mice, more cells fired at least one spike (62%, *n* = 57) and fewer cells exhibited a nonspiking phenotype in response to current injection (18.5%, *n* = 17) compared with the uninjured group (*X*^2 ^= 14.62; df = 3; *p* = 0.0022; Fig. 5*B*). A few cells fired either phasically (*n* = 9) or tonically (*n* = 9) after SCI. AHP duration was measured at 50 and 80% recovery. SCI decreased AHP_50_ (*p* < 0.003) but had no effect on AHP_80_, compared with the uninjured group (Fig. 5*C*,*D*). Lastly, despite the effects of SCI on some firing characteristics of DRG neurons, there was no increase in spontaneous firing after SCI (Fig. 5*E*,*F*), which typically indicates neuronal hyperexcitability.

Because SCI produced significant changes in RMP, neuronal firing properties, and AHP_50_, additional analyses were performed to assess the effect of 1NMP treatment on these measures. 1NMP treatment in SCI mice (SCI-1NMP) had no effect on RMP or firing properties but returned AHP_50_ values (25.3 ± 1.9 ms) to uninjured levels (27.4 ± 3.2 ms; *F*_(2,154) _= 5.8; *p* = 0.0036; ANOVA; Fig. 5*C*). This effect appears to be specific to 1NMP treatment after SCI, as in the uninjured conditions 1NMP significantly decreased AHP_50_ (refer to Table 2). Furthermore, while AHP_80_ was not significantly reduced in neurons from SCI-Veh compared with Uninjured-Veh mice, 1NMP treatment after SCI significantly increased AHP_80_ to values comparable with that seen in neurons from uninjured mice [72.1 ± 7.8 ms compared with 79.1 ± 5.8 ms (*F*_(2,156) _= 5.1; *p* = 0.0070; ANOVA; Fig. 5*D*); also see Table 2]. Interestingly, although SCI did not change AP amplitude or rheobase compared with neurons from uninjured mice, 1NMP treatment after SCI caused an increase in AP amplitude (94.3 ± 2.2 mV; *p* = 0.0303) and decreased rheobase (154.4 ± 18.2; *p* = 0.0061; refer to Table 2). No other properties were altered by SCI and/or 1NMP treatment. Altogether, these results indicate that SCI-induced changes are not mediated exclusively by TrkB signaling and furthermore, that neuronal response to TrkB agonist is probably not through altered neuronal excitability.

### Capsaicin induces inward currents in DRG neurons that are decreased after SCI

**Figure 6.**
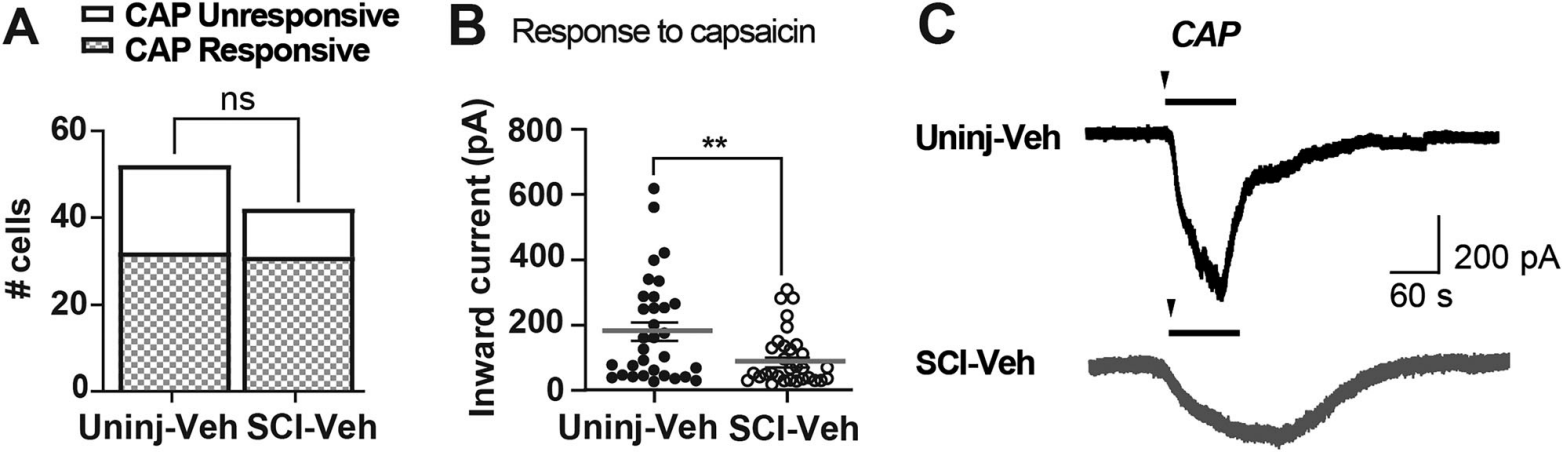
Capsaicin-induced inward currents in DRG neurons that is decreased following SCI. ***A***, There was no difference in the number of neurons responsive (checkered bar) or unresponsive (white bar) to capsaicin (CAP) between Uninjured-Veh and SCI-Veh groups (Fisher's exact test, two-tailed *p* = 0.5494). ***B***, Comparison of the peak amplitude of CAP-induced inward currents between the two groups revealed a significantly reduced inward currents after SCI (**p = 0.0096). ***C***, Representative traces of the inward currents from voltage-clamp recordings (at −60 mV) elicited by 3 µM CAP (1 min) in neurons from the Uninjured-Veh (top) and SCI-Veh (bottom) groups. Arrowheads indicate drug introduction to the bath, and the bar indicates the duration of CAP perfusion. Data points represent individual neurons; horizontal lines indicate mean ± SEM.

Most DRG neurons with small soma diameters (15–30 µm) in the DRGs are nociceptors (Lynn and Carpenter, 1982; Gold et al., 1996; Lawson, 2002; Hagenacker et al., 2005; Fang et al., 2006; Körner and Lampert, 2022), and some subpopulations of small-diameter DRG neurons are responsive to TRPV1 agonist, capsaicin. Concentration–response of capsaicin (1–30 µM) was assessed in 29 cells and showed that capsaicin evoked robust inward current responses at all concentrations but showed saturation at 3–10 µM (data not shown), similar to previous study performed in slice recordings (Liao et al., 2011). Therefore, 3 µM capsaicin was used in all the following experiments as a marker of potential nociceptors (Stucky and Lewin, 1999; Dirajlal et al., 2003; Le Pichon and Chesler, 2014). Large capsaicin currents were recorded in 63 out of 94 cells (Uninjured-Veh, *n* = 32 and SCI-Veh, *n* = 31), consistent with previously reported TRPV1-positive population (Hoffman et al., 2010; Fig. 6*A*). There was no difference in the number of capsaicin-responsiveness neurons between uninjured and SCI groups (Fisher's exact test; two-tailed *p* = 0.2710). However, the capsaicin-evoked inward currents were significantly decreased after SCI (*p* = 0.0096; Fig. 6*B*,*C*). Though such a reduction after SCI was not expected, the downward change in inward current response of both capsaicin- and 7,8-DHF-induced receptor activation could point to a potential decrease in neuronal responsivity after SCI.

### Decrease in TrkB expression in the DRGs around the lesion following SCI

**Figure 7.**
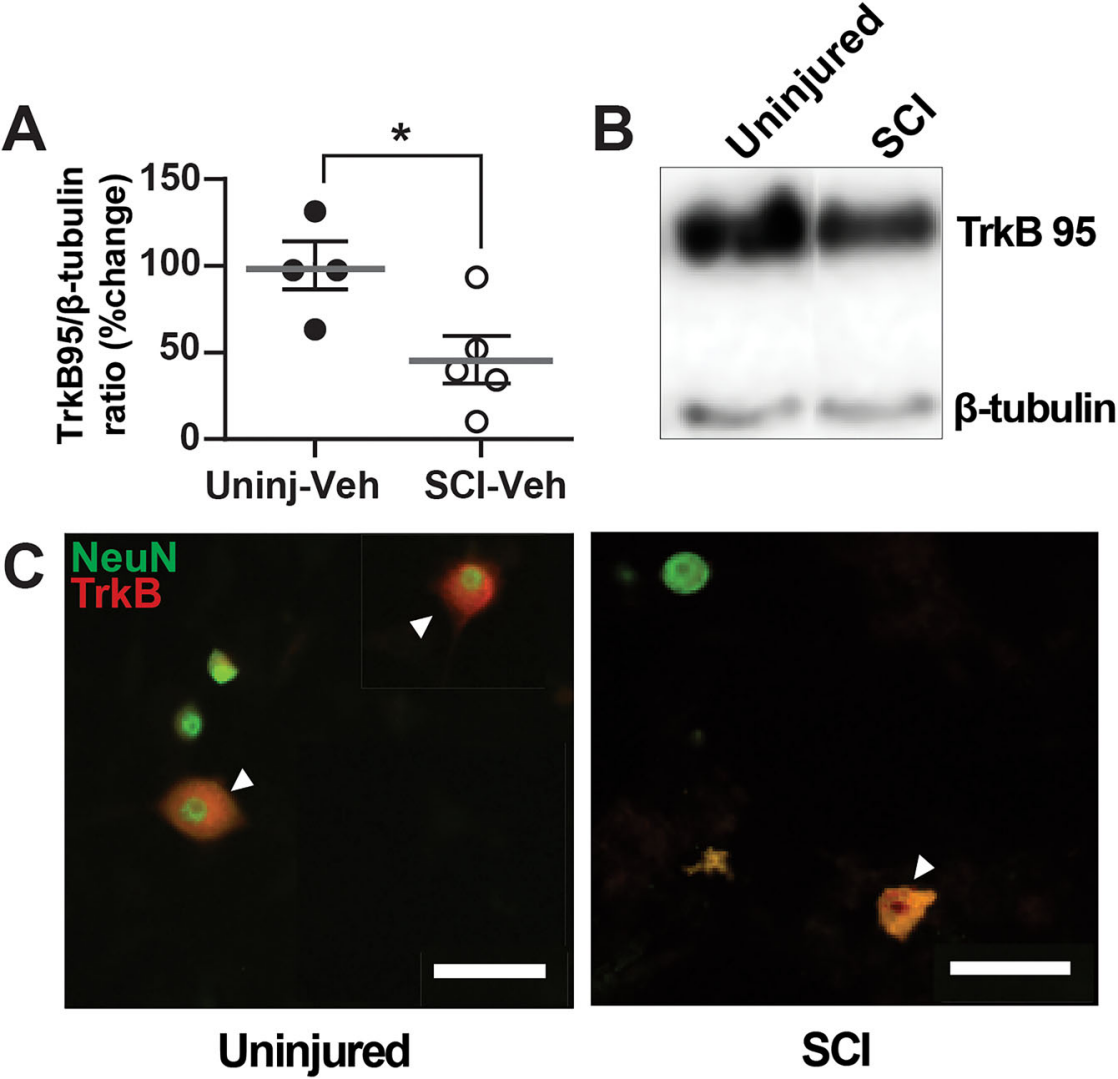
TrkB protein expression in DRG is decreased following SCI. ***A***, Histogram shows that TrkB expression in DRGs are significantly decreased 5–7 d after SCI (open circles) compared with uninjured condition (black-filled circles; *p = 0.0469). ***B***, Representative Western blot images are shown for TrkB 95 expression in the thoracic DRGs from uninjured and SCI animals (TrkB145 was not observed). ***C***, Double immunofluorescence demonstrates TrkB expression (red fluorescence) in NeuN-positive neuron (green fluorescence; merged, yellow fluorescence). Scale bars represent 50 μm. Differential pattern of colocalization (indicated by arrowheads) of TrkB and NeuN shows a potentially altered expression of cell-surface TrkB in uninjured (left) versus SCI (right) small-diameter DRG neurons.

Given the observed differences in the inward current responses in the electrophysiological studies, Western blot analyses were performed to assess potential alterations in TrkB expression in the DRGs following SCI. Western blots were processed with DRGs from spinal levels T4-L2, collected 7 d after injury. Unexpectedly, only the TrkB95 transcript was observed in the study, and its expression was decreased in the DRGs collected from SCI animals (46 ± 15% of control), compared with uninjured animals (100 ± 17%; *p* = 0.0469). The results suggest that 7 d following SCI, TrkB protein expression is decreased in the DRGs around the lesion site (Fig. 7*A*,*B*), similar to what is observed in the injured spinal cord (Garraway et al., 2011). The decrease in TrkB protein expression provides a potential mechanism for the observed profound reduction in the 7,8-DHF-induced inward current. Though not quantitated in the current study, cultured plates were stained with antibodies for TrkB and neuronal marker, NeuN, to show colocalization, which demonstrated that some small-diameter cells did indeed express TrkB and, more importantly, potential differential cell surface expression between uninjured and SCI neurons (Fig. 7*C*).

## Discussion

The present study was undertaken to examine the role of TrkB signaling in DRG neurons after SCI, and its potential contribution to pain hypersensitivity. It builds on a prior study that revealed acute maladaptive TrkB signaling contributes to pain after SCI (Martin et al., 2022). While an overwhelming number of studies have implicated BDNF and TrkB in inflammatory pain [reviewed by Garraway and Huie (2016); Garraway (2023)], notable changes that take place after SCI reveal distinct neural processes in SCI-induced neuropathic pain. For example, pain hypersensitivity that develops after SCI despite reduced expression of BDNF and TrkB in the lesioned spinal cord (Garraway et al., 2011) suggests that BDNF-TrkB signaling in the injured spinal cord does not contribute to pain after SCI. More recently, it was reported that SCI increased TrkB expression in the lumbar spinal cord of mice after the development of pain although the levels were reduced by TrkB inhibition (Martin et al., 2022). These results indicate the complexity associated with spinal BDNF and TrkB regulation of maladaptive plasticity after SCI. Further, they reveal the possibility that TrkB is involved in mechanisms that contribute to neuropathic pain after SCI that do not reside within the spinal cord, demonstrating the need for additional investigation. Here, 7,8-DHF, the selective TrkB agonist, induced large inward currents in DRG neurons from uninjured mice, which were drastically reduced after SCI. 7,8-DHF-induced currents were also significantly attenuated by treatment with 1NMP, which confirmed that the effects were mediated through TrkB signaling. Though unexpected, the observation that TrkB-mediated effects are reduced after SCI mirrors previously published results showing that BDNF-induced facilitation of afferent-evoked synaptic currents in lamina II neurons were abolished after SCI (Garraway et al., 2005). Additionally, although SCI did not induce spontaneous firing in DRG neurons as reported by Bedi et al. (2010), it altered several cell membrane properties and increased neuronal excitability, as assessed by other measures. A notable observation in this study is that TrkB protein expression in the corresponding DRGs was decreased by SCI, an observation that is similar to that reported in the lesioned spinal cord after SCI (King et al., 2000; Liebl et al., 2001; Garraway et al., 2005). Together, these results suggest that TrkB signaling is attenuated in both sensory and spinal neurons after SCI.

Sensory neurons detect and transmit peripheral stimuli and are critical for initiation of somatosensation and pain (Basbaum et al., 2009). Although their role in acute pain is well studied, less emphasis has been placed on understanding the exact role DRG neurons play in neuropathic pain, particularly after SCI, except Bedi et al. (2010), who showed that chronic hyperexcitability of nociceptors critically underlies pain after SCI [see review by K. Jang and Garraway (2024)]. Primary afferent plasticity, such as anatomical sprouting in the spinal cord, has also been implicated in pain after SCI (Detloff et al., 2016), and the release of BDNF from sensory neurons onto TrkB-expressing spinal cord dorsal horn neurons is critical to inflammatory pain (Kerr et al., 1999). Therefore, TrkB signaling was anticipated to mediate sensory neurons’ hyperexcitability as a potential underlying mechanism of neuropathic pain. Changes in several neuronal properties demonstrate that SCI produced sensory neuron hyperexcitability. For instance, SCI led to a more depolarized RMP, and more neurons fired an AP once threshold is reached. SCI decreased AHP_50_ in sensory neurons, which was restored to uninjured levels by 1NMP treatment, as was AHP_80_. AHPs play a role in determining the firing rate of neurons, where shorter AHPs result in higher firing frequencies. Calcium-activated potassium channels (K_Ca_), which underlie longer duration AHP, are important regulators of neuronal excitability (Vergara et al., 1998). Hence, this novel observation suggests that neurons are indeed more excitable after SCI, despite not displaying increased spontaneous or tonic firing. A decrease in AHP_50_ in sensory neurons is implicated in pain (Zhu et al., 2017) and supports the notion that sensory neuron hyperexcitability underlies pain after SCI. Importantly, the most substantial effect of 1NMP post-SCI was its ability to return AHP, suggesting that TrkB signaling influences activity in K_Ca_ channels after SCI. Given the complex, albeit unique, processes that underlie TrkB signaling and pain after SCI, some differential outcomes are not surprising. While all measures of neuronal hypersensitivity after SCI are not uniformly reversed by inhibiting TrkB signaling with systemic 1NMP blockade, several conclusions can be drawn.

First, neurons were identified by their sizes and response to capsaicin, thus presumed to be nociceptors. Consequently, TrkB signaling in non-nociceptive DRG neurons was not considered. Notably, the amplitude of capsaicin-induced inward currents was decreased in the recorded neurons after SCI, which suggests that overall sensitivity of small neurons in the DRG is reduced after SCI. While the apparent decrease in neuronal sensitivity is inconsistent with previous observations of TrkB-mediated pain hypersensitivity, these results suggest that TrkB signaling underlying neuropathic pain after SCI may not originate from the nociceptors. Whereas the small-diameter sensory neurons recorded in the current study are presumed to be nociceptors, DRG neurons, and even the small-diameter neurons, display substantial heterogeneity. For instance, nociceptors with cutaneous or viscera origins may have different responses after SCI. Also, according to recent RNA sequencing results, TrkB is expressed across different groups of small DRG neurons, which include C low threshold mechanoreceptors (LTMRs) and peptidergic nociceptors (Y. Zheng et al., 2019), both of which express high levels of *TrpV1*. TrkB is highly expressed in small myelinated, Aδ-LTMRs that are less responsive to capsaicin (Abraira and Ginty, 2013). An earlier study also reported a substantial increase of BDNF in large-diameter TrkB expressing DRG neurons following axotomy. However, BDNF expression in small neurons were unchanged (Michael et al., 1999), suggesting that TrkB signaling in large DRG neurons might contribute to pain after injury. These observations provide likely scenarios for TrkB to influence nociception by DRG processes that are independent of nociceptors. However, appropriate measures to identify and target TrkB-expressing subpopulations in the DRGs, such as Aδ-LTMRs and large-diameter sensory neurons, would be required to further uncover TrkB's contribution in pain signaling that is not limited to nociceptors.

Second, the locus of TrkB-mediated pain hypersensitivity may be more peripheral than the DRGs. After SCI, BDNF and TrkB (unpublished observation) and phosphorylated extracellular signal-regulated kinase (pERK; Martin et al., 2022) are increased in the trunk skin at the level of the injury. Because pERK is activated downstream of TrkB, its upregulation could indicate a potential peripheral mechanism for contribution of TrkB signaling in pain. The skin is innervated by heterogeneous populations of sensory neurons, such as D-hairs (L. Li et al., 2011) and Aβ-mechanoreceptors (Michael et al., 1999), which show high sensitivity among cutaneous mechanoreceptors and respond to dynamic stimuli. TrkB expression has been identified in these sensory afferents, and BDNF-TrkB signaling in the skin was shown to be required for normal mechanotransduction (Rutlin et al., 2014). Hence, impaired BDNF-TrkB signaling in the skin could alter the function of these mechanoreceptors, leading to the development of mechanical allodynia. TrkB ablation in the D-hair, Aβ mechanoreceptors, and Aδ mechanoreceptors reduced neuropathic pain behavior after contusion SCI in rodents (Sliwinski et al., 2024), suggesting that mechanoreceptors are also likely involved in the initiation of neuropathic pain. Therefore, it can be assumed that conversely, overexpression of BDNF and TrkB in the skin leads to increased pain. Consistent with the notion that peripheral afferents signal pain after SCI, C-LTMRs, which are a subpopulation of cutaneous afferents that normally signal emotional touch, were shown signal pain after SCI (Noble et al., 2022). Additionally, neurotrophins also play an important role in the non-neuronal tissues, such as mast cells (Kawamoto et al., 2002) and endothelial cells (Raychaudhuri et al., 2001). Keratinocytes also release BDNF (Bronzetti et al., 1995), albeit in low amounts (Terracina et al., 2003). Therefore, both afferent plasticity and changes in endogenous BDNF and TrkB in the skin can critically underlie pain after SCI in a manner distinct from the DRGs or the spinal cord.

Clearly, the exact site of maladaptive TrkB signaling that leads to pain is impossible to pinpoint, as 1NMP was administered systemically. While the likelihood of a peripheral site of action is supported, as discussed above, spinal TrkB mechanisms cannot be completely excluded. Despite many reports of TrkB expression being decreased in the lesioned spinal cord, Martin et al. (2022) showed SCI increased TrkB in the lumbar spinal cord, which was attenuated by 1NMP treatment. Interestingly, in the same study, TrkB inhibition effectively blocked hindpaw hypersensitivity but had no effect on at-level aversive pain, suggesting that SCI differentially impacts TrkB signaling in the spinal cord. Similarly, DRGs innervate tissues that are associated with local body segments. DRGs from different vertebral segments have different innervation (e.g., thoracic DRGs innervate the trunk while the lumbar DRGs innervate the lower body; Haberberger et al., 2019; Noseda et al., 2019), morphology (Hasegawa et al., 1996), and composition [i.e., thoracic DRGs had the highest proportion of C-LTMRs (Jung et al., 2023)] profiles, demonstrating the possibility that changes in TrkB could have regionally distinct effects after SCI. Because this study focused primarily on activity in thoracic DRG neurons adjacent to the injury, assessment of lumbar DRGs could yield different outcomes.

Third, these complex results reveal a potential multifaceted nature of the changes in TrkB expression. While the current study showed a decrease in TrkB expression in the DRG after SCI, conclusions about TrkB synthesis, internalization, or cell surface expression cannot be made. After SCI, there is an increasingly inhibitory environment established around the injury site (Meyer-Franke et al., 1998), including a sustained decrease in external modulators, such as cyclic adenosine monophosphate (cAMP), in the spinal cord and surrounding tissues (Meyer-Franke et al., 1998). cAMP has been identified to regulate TrkB cell surface expression (Boulanger and Poo, 1999; Tartaglia et al., 2001) and phosphorylation (Schwab and Bartholdi, 1996; Fawcett and Asher, 1999; Silver and Miller, 2004), by rapid TrkB surface transport from the pre-existing intracellular source, instead of de novo transcription or translation of the receptor mRNA. Given the proximity to the injury, neighboring DRGs are expected to undergo such changes. Thus, a decrease in TrkB transport to the membrane of sensory neurons likely leads to reduced exposure to the agonist. Indeed, studies have shown that even with the robust overall expression levels of TrkB, low cell surface expression in cultured neurons have resulted in low responsiveness to BDNF (Ghosh et al., 2012). Because 7,8-DHF has been shown to mimic BDNF's neurotrophic signaling and TrkB activation, it may also bind similarly to TrkB in cultured dissociated DRG neurons but lead to low responsiveness if cell surface expression of TrkB is decreased after injury. Altered TrkB cell surface expression and regulation of endogenous BDNF could lead to changes in both the TrkB-mediated neuronal responses and overall TrkB expression levels. Although not quantitated in the current study, some differential patterns of fluorescent labeling in TrkB were noted between cultured uninjured and SCI neurons, indicating a potential mechanism. Therefore, an investigation of steady-state levels of TrkB or specific cell surface expression of TrkB in nociceptors after SCI is needed to further understand the implications of electrophysiological changes reported.

Like BDNF, neurotrophin (NT)-4 binds TrkB with high affinity (Klein et al., 1992). Although NT-4-TrkB signaling is implicated in the survival of different types of sensory neurons (J. L. Zheng et al., 1995; Stucky et al., 1998) in the mature system, NT-4 is expressed predominantly by motor neurons in the ventral horn of the spinal cord and does not significantly contribute to nociceptive or neuropathic pain (Funakoshi et al., 1993; Buck et al., 2000; Heppenstall and Lewin, 2001; Yajima et al., 2005). Given the limited role of NT-4 in pain pathways, the current study infers TrkB signaling in DRG neurons are driven by BDNF, which is also altered in the DRG and spinal cord after SCI (Hougland et al., 2012). Nonetheless, a potential limited role of NT-4 in neuronal excitability after SCI cannot be ignored.

Fourth, while there are no identified endogenous inhibitor of TrkB, truncated TrkB isoform TrkB.T1 is considered a potential inhibitor of the full-length receptor by ligand trapping or acting as a dominant-negative receptor (Biffo et al., 1995; Eide et al., 1996; Y-X. Li et al., 1998; Palko et al., 1999). Both spinal and peripheral TrkB are composed of different TrkB isoforms. The two major isoforms include full-length TrkB (TrkB.FL) and truncated TrkB (TrkB.T1/T2) forms. Following SCI, truncated forms of TrkB receptor were shown to be upregulated in the spinal cord at the level of the injury (D'Arcangelo et al., 1993; Hilborn et al., 1998), playing an important role in SCI-induced pain through regulation of cell cycle pathways (Wu et al., 2013). Inhibition of TrkB.T1 was also shown to reduce inflammation and improve motor function and symptoms of neuropathic pain after SCI (Matyas et al., 2017). In the DRGs, Lee et al. (1999) reported that mRNA of truncated TrkB receptor increases in inflammatory pain models but not full-length TrkB, and the truncated TrkB increased in the lumbar DRGs in a model of chronic neuropathic pain (Wei et al., 2021). Although in this study we report changes in the truncated form, SCI can result in changes to the ratio of TrkB.FL and truncated, inactive isoforms in the thoracic DRGs as well to ultimately affect the inward current response and change the overall expression levels.

Lastly, TrkB, as a receptor tyrosine kinase, may alter nociceptor sensitivity after SCI through biochemical pathways that ultimately result in changes in gene expression and synaptic strength or through coordination with other ion channels such as VGSCs, K_Ca_, or voltage-gated calcium channels (e.g., Ca_v_ 3.2). Both SCI and 1NMP treatment after SCI modified AHP duration, suggesting that TrkB interacts with K_Ca_ after SCI although similar changes were not seen in uninjured condition. Also, SCI did not change the onset of 7,8-DHF-induced inward currents which confirm that the responses were most likely mediated by TrkB. However, the changes in the latency to peak and amplitude of inward current may reflect modulatory activity through changes in expression or sensitivity of other ion channels and not merely due to the decreased expression of TrkB. For instance, VGSCs have been investigated thoroughly for their role in setting the AP threshold and maximum firing frequency. VGSCs can also be modulated by receptor tyrosine kinases (D'Arcangelo et al., 1993; Hilborn et al., 1998). Specifically, some subtypes of VGSCs, such as Na_v_ 1.7, 1.8, and 1.9, that are highly or exclusively expressed in DRG neurons (Rush et al., 1998, 2007) have been found to be functionally associated with TrkB (Blum et al., 2002) as well as neuropathic pain. Therefore, interactions between VGSC (or other channels) and TrkB are expected to be altered following SCI, but further studies are required to ascertain the impact of these interactions on TrkB signaling and pain hypersensitivity after SCI. For example, a pharmacological investigation into changes in inward current responses with Na_v_ subtype-specific ligands would provide more insight into the role of VGSC in TrkB signaling and plasticity observed in small-diameter neurons after SCI.

Several additional mediators that are not directly addressed in this study can play a role in neuronal hyperexcitability and pain after SCI. For example, as this study was done in SCI and uninjured mice, only SCI mice were treated with meloxicam, the cyclooxygenase-2 inhibitor. Because prior behavioral studies revealed acute treatment with meloxicam immediately after SCI does not abrogate SCI-induced pain hypersensitivity (Martin et al., 2022; Noble et al., 2022), it is not expected that meloxicam will reduce sensory neuron excitability, although the possibility exists. Also, it was previously shown that meloxicam's levels in plasma are drastically reduced by 12 h after treatment (P. H. Chen et al., 2016), further suggesting that its effects in this study are expected to be non-essential. Future studies will more thoroughly examine the myriads of interactions that can influence TrkB signaling in sensory neuron dysfunction or pain hypersensitivity following SCI.

Overall, the current study introduced a mechanistic insight into the role of peripheral TrkB signaling in pain hypersensitivity after SCI, focusing on presumed nociceptive DRG neurons. The results strongly suggest that nociceptor excitability and TrkB signaling are distinctly impacted by SCI, where an increase in neuronal excitability is accompanied by a decrease in TrkB agonist-induced current, probably due to a decrease in TrkB expression in the DRGs. Nonetheless, a notable limitation of our study is that the small-diameter neurons in the electrophysiological recordings are not ascertained to be nociceptors. As stated above, TrkB expression in DRG is not limited to small nociceptors but also includes medium- to large-diameter DRG neurons (Vega et al., 1994; Anand et al., 1997; Widenfalk et al., 1999), as well as touch encoding neurons. To fully elucidate the role of TrkB signaling in DRG neurons in pain hypersensitivity, future studies that enable specific targeting of TrkB (e.g., transgenic mice that allow visual confirmation of TrkB expression) and nociceptors are needed. Additionally, although these studies were performed in a transgenic mouse that allows selective TrkB inhibition, an important concern is whether the expression of the mutated TrkB in F616 mice fully recapitulates that of native TrkB. This concern is somewhat allayed by a previous study showing similar formalin-induced responses in wild-type and F616 mice (Martin et al., 2022). Yet, additional studies in wild-type mice will be helpful to profile changes in native TrkB signaling after SCI. Lastly, because sensory neurons are sensitive to the dissociation process (Owen and Egerton, 2012), the enzymatic and mechanical dissociation and the 24 h incubation period may have allowed an altered phenotype to be established in neurons, thereby changing their properties. However, because these conditions were consistent across all experimental groups, the observed changes in neuronal responses are more likely induced by SCI and 1NMP treatments rather than an altered phenotype. Despite these limitations, we present novel evidence that suggests SCI-induced maladaptive plasticity in sensory neurons is sensitive to TrkB-mediated procession. Nonetheless, until more studies are done, we conclude that TrkB signaling is implicated in neuropathic pain after SCI but the exact neural mechanism, potentially requiring a collusion of spinal and peripheral plasticity, remains to be determined.

## Synthesis

Reviewing Editor: Benjamin Deneen, Baylor College of Medicine Decisions are customarily a result of the Reviewing Editor and the peer reviewers coming together and discussing their recommendations until a consensus is reached. When revisions are invited, a fact-based synthesis statement explaining their decision and outlining what is needed to prepare a revision will be listed below. The following reviewer(s) agreed to reveal their identity: NONE.

The authors have done a commendable job in responding to the reviewer comments and have significantly improved the depth, breadth, and overall quality of this manuscript.

## References

[B1] Abraira VE, Ginty DD (2013) The sensory neurons of touch. Neuron 79:618–639. 10.1016/j.neuron.2013.07.051 23972592 PMC3811145

[B2] Anand P, Terenghi G, Birch R, Wellmer A, Cedarbaum JM, Lindsay RM, Williams-Chestnut RE, Sinicropi DV (1997) Endogenous NGF and CNTF levels in human peripheral nerve injury. Neuroreport 8:1935–1938. 10.1097/00001756-199705260-00028 9223080

[B3] Basbaum AI, Bautista DM, Scherrer G, Julius D (2009) Cellular and molecular mechanisms of pain. Cell 139:267–284. 10.1016/j.cell.2009.09.028 19837031 PMC2852643

[B4] Basso DM, Fisher LC, Anderson AJ, Jakeman LB, McTigue DM, Popovich PG (2006) Basso mouse scale for locomotion detects differences in recovery after spinal cord injury in five common mouse strains. J Neurotrauma 23:635–659. 10.1089/neu.2006.23.635 16689667

[B5] Bedi SS, Yang Q, Crook RJ, Du J, Wu Z, Fishman HM, Grill RJ, Carlton SM, Walters ET (2010) Chronic spontaneous activity generated in the somata of primary nociceptors is associated with pain-related behavior after spinal cord injury. J Neurosci 30:14870–14882. 10.1523/JNEUROSCI.2428-10.2010 21048146 PMC3073589

[B6] Biffo S, Offenhäuser N, Carter BD, Barde Y-A (1995) Selective binding and internalisation by truncated receptors restrict the availability of BDNF during development. Development 121:2461–2470. 10.1242/dev.121.8.2461 7671810

[B7] Blum R, Kafitz KW, Konnerth A (2002) Neurotrophin-evoked depolarization requires the sodium channel Na(V)1.9. Nature 419:687–693. 10.1038/nature01085 12384689

[B8] Boulanger L, Poo MM (1999) Gating of BDNF-induced synaptic potentiation by cAMP. Science 284:1982–1984. 10.1126/science.284.5422.1982 10373115

[B9] Bronzetti E, Ciriaco E, Germanà G, Vega JA (1995) Immunohistochemical localization of neurotrophin receptor proteins in human skin. Ital J Anat Embryol 100:565–571. 11322337

[B10] Buck CR, Seburn KL, Cope TC (2000) Neurotrophin expression by spinal motoneurons in adult and developing rats. J Comp Neurol 416:309–318. 10.1002/(sici)1096-9861(20000117)416:3<309::aid-cne3>3.0.co;2-u 10602090

[B11] Cardenas CG, Mar LPD, Scroggs RS (1995) Variation in serotonergic inhibition of calcium channel currents in four types of rat sensory neurons differentiated by membrane properties. J Neurophysiol 74:1870–1879. 10.1152/jn.1995.74.5.1870 8592180

[B12] Caterina MJ, Schumacher MA, Tominaga M, Rosen TA, Levine JD, Julius D (1997) The capsaicin receptor: a heat-activated ion channel in the pain pathway. Nature 389:816–824. 10.1038/39807 9349813

[B13] Cazorla M, Prémont J, Mann A, Girard N, Kellendonk C, Rognan D (2011) Identification of a low-molecular weight TrkB antagonist with anxiolytic and antidepressant activity in mice. J Clin Invest 121:1846–1857. 10.1172/jci43992 21505263 PMC3083767

[B14] Chen PH, Boyd KL, Fickle EK, Locuson CW (2016) Subcutaneous meloxicam suspension pharmacokinetics in mice and dose considerations for postoperative analgesia. J Vet Pharmacol Ther 39:356–362. 10.1111/jvp.12297 26896236 PMC7166601

[B15] Chen X, Ye H, Kuruvilla R, Ramanan N, Scangos KW, Zhang C, Johnson NM, England PM, Shokat KM, Ginty DD (2005) A chemical-genetic approach to studying neurotrophin signaling. Neuron 46:13–21. 10.1016/j.neuron.2005.03.009 15820690

[B16] Cho HJ, Kim JK, Park HC, Kim JK, Kim DS, Ha SO, Hong HS (1998) Changes in brain-derived neurotrophic factor immunoreactivity in rat dorsal root ganglia, spinal cord, and gracile nuclei following cut or crush injuries. Exp Neurol 154:224–230. 10.1006/exnr.1998.6936 9875283

[B17] D'Arcangelo G, Paradiso K, Shepherd D, Brehm P, Halegoua S, Mandel G (1993) Neuronal growth factor regulation of two different sodium channel types through distinct signal transduction pathways. J Cell Biol 122:915–921. 10.1083/jcb.122.4.915 8394370 PMC2119579

[B18] Davidson S, Copits BA, Zhang J, Page G, Ghetti A, Gereau RW 4th (2014) Human sensory neurons: membrane properties and sensitization by inflammatory mediators. Pain 155:1861–1870. 10.1016/j.pain.2014.06.017 24973718 PMC4158027

[B19] Detloff MR, et al. (2016) Delayed exercise is ineffective at reversing aberrant nociceptive afferent plasticity or neuropathic pain after spinal cord injury in rats. Neurorehabil Neural Repair 30:685–700. 10.1177/1545968315619698 26671215 PMC4907889

[B20] Dhandapani R, et al. (2018) Control of mechanical pain hypersensitivity in mice through ligand-targeted photoablation of TrkB-positive sensory neurons. Nat Commun 9:1640. 10.1038/s41467-018-04049-3 29691410 PMC5915601

[B21] Dirajlal S, Pauers LE, Stucky CL (2003) Differential response properties of IB(4)-positive and -negative unmyelinated sensory neurons to protons and capsaicin. J Neurophysiol 89:513–524. 10.1152/jn.00371.2002 12522198

[B22] Eide FF, Vining ER, Eide BL, Zang K, Wang XY, Reichardt LF (1996) Naturally occurring truncated trkB receptors have dominant inhibitory effects on brain-derived neurotrophic factor signaling. J Neurosci 16:3123–3129. 10.1523/jneurosci.16-10-03123.1996 8627351 PMC2710135

[B23] Ernfors P, Rosario CM, Merlio JP, Grant G, Aldskogius H, Persson H (1993) Expression of mRNAs for neurotrophin receptors in the dorsal root ganglion and spinal cord during development and following peripheral or central axotomy. Brain Res Mol Brain Res 17:217–226. 10.1016/0169-328x(93)90005-a 8510496

[B24] Fang X, Djouhri L, McMullan S, Berry C, Waxman SG, Okuse K, Lawson SN (2006) Intense isolectin-B4 binding in rat dorsal root ganglion neurons distinguishes C-fiber nociceptors with broad action potentials and high Nav1.9 expression. J Neurosci 26:7281–7292. 10.1523/jneurosci.1072-06.2006 16822986 PMC6673936

[B25] Fawcett JW, Asher RA (1999) The glial scar and central nervous system repair. Brain Res Bull 49:377–391. 10.1016/S0361-9230(99)00072-6 10483914

[B26] Felix ER, Cardenas DD, Bryce TN, Charlifue S, Lee TK, MacIntyre B, Mulroy S, Taylor H (2022) Prevalence and impact of neuropathic and nonneuropathic pain in chronic spinal cord injury. Arch Phys Med Rehabil 103:729–737. 10.1016/j.apmr.2021.06.022 34343523

[B27] Fukuoka T, Kondo E, Dai Y, Hashimoto N, Noguchi K (2001) Brain-derived neurotrophic factor increases in the uninjured dorsal root ganglion neurons in selective spinal nerve ligation model. J Neurosci 21:4891–4900. 10.1523/JNEUROSCI.21-13-04891.2001 11425916 PMC6762362

[B28] Funakoshi H, Frisén J, Barbany G, Timmusk T, Zachrisson O, Verge VM, Persson H (1993) Differential expression of mRNAs for neurotrophins and their receptors after axotomy of the sciatic nerve. J Cell Biol 123:455–465. 10.1083/jcb.123.2.455 8408225 PMC2119843

[B29] Galvao J, Davis B, Tilley M, Normando E, Duchen MR, Cordeiro MF (2014) Unexpected low-dose toxicity of the universal solvent DMSO. FASEB J 28:1317–1330. 10.1096/fj.13-235440 24327606

[B30] Garraway SM (2023) *BDNF-induced plasticity of spinal circuits underlying pain and learning*. New York: Oxford University Press.

[B31] Garraway SM, Anderson AJ, Mendell LM (2005) BDNF-induced facilitation of afferent-evoked responses in lamina II neurons is reduced after neonatal spinal cord contusion injury. J Neurophysiol 94:1798–1804. 10.1152/jn.00179.2005 15901762

[B32] Garraway SM, Huie JR (2016) Spinal plasticity and behavior: BDNF-induced neuromodulation in uninjured and injured spinal cord. Neural Plast 2016:9857201. 10.1155/2016/9857201 27721996 PMC5046018

[B33] Garraway SM, Mendell LM (2007) Spinal cord transection enhances afferent-evoked inhibition in lamina II neurons and abolishes BDNF-induced facilitation of their sensory input. J Neurotrauma 24:379–390. 10.1089/neu.2006.0115 17376001

[B34] Garraway SM, Turtle JD, Huie JR, Lee KH, Hook MA, Woller SA, Grau JW (2011) Intermittent noxious stimulation following spinal cord contusion injury impairs locomotor recovery and reduces spinal brain-derived neurotrophic factor-tropomyosin-receptor kinase signaling in adult rats. Neuroscience 199:86–102. 10.1016/j.neuroscience.2011.10.007 22027236 PMC3237800

[B35] Ghosh M, Garcia-Castillo D, Aguirre V, Golshani R, Atkins CM, Bramlett HM, Dietrich WD, Pearse DD (2012) Proinflammatory cytokine regulation of cyclic AMP-phosphodiesterase 4 signaling in microglia in vitro and following CNS injury. Glia 60:1839–1859. 10.1002/glia.22401 22865690 PMC4383287

[B36] Gold MS, Dastmalchi S, Levine JD (1996) Co-expression of nociceptor properties in dorsal root ganglion neurons from the adult rat in vitro. Neuroscience 71:265–275. 10.1016/0306-4522(95)00433-5 8834408

[B37] Ha SO, Kim JK, Hong HS, Kim DS, Cho HJ (2001) Expression of brain-derived neurotrophic factor in rat dorsal root ganglia, spinal cord and gracile nuclei in experimental models of neuropathic pain. Neuroscience 107:301–309. 10.1016/s0306-4522(01)00353-0 11731104

[B38] Haberberger RV, Barry C, Dominguez N, Matusica D Human dorsal root ganglia [Review]. Front Cell Neurosci 13:271. 10.3389/fncel.2019.00271 31293388 PMC6598622

[B39] Hagenacker T, Splettstoesser F, Greffrath W, Treede RD, Büsselberg D (2005) Capsaicin differentially modulates voltage-activated calcium channel currents in dorsal root ganglion neurones of rats. Brain Res 1062:74–85. 10.1016/j.brainres.2005.09.033 16269136

[B40] Hajebrahimi Z, Mowla SJ, Movahedin M, Tavallaei M (2008) Gene expression alterations of neurotrophins, their receptors and prohormone convertases in a rat model of spinal cord contusion. Neurosci Lett 441:261–266. 10.1016/j.neulet.2008.06.046 18585435

[B41] Hasegawa T, Mikawa Y, Watanabe R, An HS (1996) Morphometric analysis of the lumbosacral nerve roots and dorsal root ganglia by magnetic resonance imaging. Spine 21:1005–1009. 10.1097/00007632-199605010-00001 8724082

[B42] Heppenstall PA, Lewin GR (2001) BDNF but not NT-4 is required for normal flexion reflex plasticity and function. Proc Natl Acad Sci U S A 98:8107–8112. 10.1073/pnas.141015098 11438749 PMC35475

[B43] Hilborn MD, Vaillancourt RR, Rane SG (1998) Growth factor receptor tyrosine kinases acutely regulate neuronal sodium channels through the src signaling pathway. J Neurosci 18:590–600. 10.1523/jneurosci.18-02-00590.1998 9425001 PMC6792527

[B44] Hochman S, Garraway SM, Pockett S (1997) Membrane properties of deep dorsal horn neurons from neonatal rat spinal cord in vitro. Brain Res 767:214–219. 10.1016/S0006-8993(97)00578-7 9367250

[B45] Hoffman EM, Schechter R, Miller KE (2010) Fixative composition alters distributions of immunoreactivity for glutaminase and two markers of nociceptive neurons, Nav1.8 and TRPV1, in the rat dorsal root ganglion. J Histochem Cytochem 58:329–344. 10.1369/jhc.2009.954008 20026672 PMC2842596

[B46] Hougland MT, Harrison BJ, Magnuson DS, Rouchka EC, Petruska JC (2012) The transcriptional response of neurotrophins and their tyrosine kinase receptors in lumbar sensorimotor circuits to spinal cord contusion is affected by injury severity and survival time. Front Physiol 3:478. 10.3389/fphys.2012.00478 23316162 PMC3540763

[B47] Hu X, et al. (2023) A TRPV4-dependent neuroimmune axis in the spinal cord promotes neuropathic pain. J Clin Invest 133:1–16. 10.1172/JCI161507 36701202 PMC9974096

[B48] Hulsebosch CE (2002) Recent advances in pathophysiology and treatment of spinal cord injury. Adv Physiol Educ 26:238–255. 10.1152/advan.00039.2002 12443996

[B49] Hulsebosch CE, Hains BC, Crown ED, Carlton SM (2009) Mechanisms of chronic central neuropathic pain after spinal cord injury. Brain Res Rev 60:202–213. 10.1016/j.brainresrev.2008.12.010 19154757 PMC2796975

[B50] Jang S-W, et al. (2010) A selective TrkB agonist with potent neurotrophic activities by 7,8-dihydroxyflavone. Proc Natl Acad Sci U S A 107:2687–2692. 10.1073/pnas.0913572107 20133810 PMC2823863

[B51] Jang K, Garraway SM (2024) A review of dorsal root ganglia and primary sensory neuron plasticity mediating inflammatory and chronic neuropathic pain. Neurobiol Pain 15:100151. 10.1016/j.ynpai.2024.100151 38314104 PMC10837099

[B52] Jung M, Dourado M, Maksymetz J, Jacobson A, Laufer BI, Baca M, Foreman O, Hackos DH, Riol-Blanco L, Kaminker JS (2023) Cross-species transcriptomic atlas of dorsal root ganglia reveals species-specific programs for sensory function. Nat Commun 14:366. 10.1038/s41467-023-36014-0 36690629 PMC9870891

[B53] Kawamoto K, Aoki J, Tanaka A, Itakura A, Hosono H, Arai H, Kiso Y, Matsuda H (2002) Nerve growth factor activates mast cells through the collaborative interaction with lysophosphatidylserine expressed on the membrane surface of activated platelets. J Immunol 168:6412–6419. 10.4049/jimmunol.168.12.6412 12055260

[B54] Kerr BJ, Bradbury EJ, Bennett DL, Trivedi PM, Dassan P, French J, Shelton DB, McMahon SB, Thompson SW (1999) Brain-derived neurotrophic factor modulates nociceptive sensory inputs and NMDA-evoked responses in the rat spinal cord. J Neurosci 19:5138–5148. 10.1523/JNEUROSCI.19-12-05138.1999 10366647 PMC6782650

[B55] King VR, Bradbury EJ, McMahon SB, Priestley JV (2000) Changes in truncated trkB and p75 receptor expression in the rat spinal cord following spinal cord hemisection and spinal cord hemisection plus neurotrophin treatment. Exp Neurol 165:327–341. 10.1006/exnr.2000.7480 10993692

[B56] Klein R, Lamballe F, Bryant S, Barbacid M (1992) The trkB tyrosine protein kinase is a receptor for neurotrophin-4. Neuron 8:947–956. 10.1016/0896-6273(92)90209-V 1375038

[B57] Koerber HR, Druzinsky RE, Mendell LM (1988) Properties of somata of spinal dorsal root ganglion cells differ according to peripheral receptor innervated. J Neurophysiol 60:1584–1596. 10.1152/jn.1988.60.5.1584 3199173

[B58] Körner J, Lampert A (2022) Functional subgroups of rat and human sensory neurons: a systematic review of electrophysiological properties. Pflügers Arch 474:367–385. 10.1007/s00424-021-02656-6 35031856 PMC8924089

[B59] Lawson SN (2002) Phenotype and function of somatic primary afferent nociceptive neurones with C-, Aδ- or Aα/β-fibres. Exp Physiol 87:239–244. 10.1113/eph8702350 29345433

[B60] Lawson SN, McCarthy PW, Prabhakar E (1996) Electrophysiological properties of neurones with CGRP-like immunoreactivity in rat dorsal root ganglia. J Comp Neurol 365:355–366. 10.1002/(sici)1096-9861(19960212)365:3<355::Aid-cne2>3.0.Co;2-3 8822175

[B61] Lee SL, Kim JK, Kim DS, Cho HJ (1999) Expression of mRNAs encoding full-length and truncated TrkB receptors in rat dorsal root ganglia and spinal cord following peripheral inflammation. Neuroreport 10:2847–2851. 10.1097/00001756-199909090-00027 10511451

[B62] Le Pichon CE, Chesler AT (2014) The functional and anatomical dissection of somatosensory subpopulations using mouse genetics. Front Neuroanat 8:21. 10.3389/fnana.2014.00021 24795573 PMC4001001

[B63] Li L, et al. (2011) The functional organization of cutaneous low-threshold mechanosensory neurons. Cell 147:1615–1627. 10.1016/j.cell.2011.11.027 22196735 PMC3262167

[B64] Li Y-X, Xu Y, Ju D, Lester HA, Davidson N, Schuman EM (1998) Expression of a dominant negative TrkB receptor, T1, reveals a requirement for presynaptic signaling in BDNF-induced synaptic potentiation in cultured hippocampal neurons. Proc Natl Acad Sci U S A 95:10884–10889. 10.1073/pnas.95.18.10884 9724799 PMC27990

[B65] Liao HT, Lee HJ, Ho YC, Chiou LC (2011) Capsaicin in the periaqueductal gray induces analgesia via metabotropic glutamate receptor-mediated endocannabinoid retrograde disinhibition. Br J Pharmacol 163:330–345. 10.1111/j.1476-5381.2011.01214.x 21232043 PMC3087135

[B66] Liebl DJ, Huang W, Young W, Parada LF (2001) Regulation of Trk receptors following contusion of the rat spinal cord. Exp Neurol 167:15–26. 10.1006/exnr.2000.7548 11161589

[B67] Liu C, Chan CB, Ye K (2016) 7,8-dihydroxyflavone, a small molecular TrkB agonist, is useful for treating various BDNF-implicated human disorders. Transl Neurodegener 5:2. 10.1186/s40035-015-0048-7 26740873 PMC4702337

[B68] Lynn B, Carpenter SE (1982) Primary afferent units from the hairy skin of the rat hind limb. Brain Res 238:29–43. 10.1016/0006-8993(82)90768-5 6282398

[B69] Mantilla CB, Stowe JM, Sieck DC, Ermilov LG, Greising SM, Zhang C, Shokat KM, Sieck GC (2014) Trkb kinase activity maintains synaptic function and structural integrity at adult neuromuscular junctions. J Appl Physiol 117:910–920. 10.1152/japplphysiol.01386.2013 25170066 PMC4199990

[B70] Martin KK, Noble DJ, Parvin S, Jang K, Garraway SM (2022) Pharmacogenetic inhibition of TrkB signaling in adult mice attenuates mechanical hypersensitivity and improves locomotor function after spinal cord injury. Front Cell Neurosci 16:987236. 10.3389/fncel.2022.987236 36226073 PMC9548551

[B71] Matyas JJ, O'Driscoll CM, Yu L, Coll-Miro M, Daugherty S, Renn CL, Faden AI, Dorsey SG, Wu J (2017) Truncated TrkB. T1-mediated astrocyte dysfunction contributes to impaired motor function and neuropathic pain after spinal cord injury. J Neurosci 37:3956–3971. 10.1523/JNEUROSCI.3353-16.2017 28270575 PMC5394902

[B72] Merighi A, Salio C, Ghirri A, Lossi L, Ferrini F, Betelli C, Bardoni R (2008) BDNF as a pain modulator. Prog Neurobiol 85:297–317. 10.1016/j.pneurobio.2008.04.004 18514997

[B73] Meyer-Franke A, Wilkinson GA, Kruttgen A, Hu M, Munro E, Hanson MG Jr, Reichardt LF, Barres BA (1998) Depolarization and cAMP elevation rapidly recruit TrkB to the plasma membrane of CNS neurons. Neuron 21:681–693. 10.1016/s0896-6273(00)80586-3 9808456 PMC2693071

[B74] Michael GJ, Averill S, Nitkunan A, Rattray M, Bennett DL, Yan Q, Priestley JV (1997) Nerve growth factor treatment increases brain-derived neurotrophic factor selectively in TrkA-expressing dorsal root ganglion cells and in their central terminations within the spinal cord. J Neurosci 17:8476–8490. 10.1523/JNEUROSCI.17-21-08476.1997 9334420 PMC6573719

[B75] Michael GJ, Averill S, Shortland PJ, Yan Q, Priestley JV (1999) Axotomy results in major changes in BDNF expression by dorsal root ganglion cells: BDNF expression in large trkB and trkC cells, in pericellular baskets, and in projections to deep dorsal horn and dorsal column nuclei. Eur J Neurosci 11:3539–3551. 10.1046/j.1460-9568.1999.00767.x 10564362

[B76] Noble DJ, Dongmo R, Parvin S, Martin KK, Garraway SM (2022) C-low threshold mechanoreceptor activation becomes sufficient to trigger affective pain in spinal cord-injured mice in association with increased respiratory rates. Front Integr Neurosci 16:1081172. 10.3389/fnint.2022.1081172 36619238 PMC9811591

[B77] Noseda R, Melo-Carrillo A, Nir R-R, Strassman AM, Burstein R (2019) Non-trigeminal nociceptive innervation of the posterior dura: implications to occipital headache. J Neurosci 39:1867–1880. 30622169 10.1523/JNEUROSCI.2153-18.2018 PMC6407291

[B78] Obata K, Noguchi K (2006) BDNF in sensory neurons and chronic pain. Neurosci Res 55:1–10. 10.1016/j.neures.2006.01.005 16516994

[B79] Owen DE, Egerton J (2012) Culture of dissociated sensory neurons from dorsal root ganglia of postnatal and adult rats. In: *Neurotrophic factors: methods and protocols* (Skaper SD, ed), pp 179–187. Totowa: Humana Press. 10.1007/978-1-61779-536-7_16 22367811

[B80] Palko ME, Coppola V, Tessarollo L (1999) Evidence for a role of truncated trkC receptor isoforms in mouse development. J Neurosci 19:775–782. 10.1523/JNEUROSCI.19-02-00775.1999 9880597 PMC6782202

[B81] Pareja-Cajiao M, Gransee HM, Cole NA, Sieck GC, Mantilla CB (2020) Inhibition of TrkB kinase activity impairs transdiaphragmatic pressure generation. J Appl Physiol 128:338–344. 10.1152/japplphysiol.00564.2019 31944892 PMC7052584

[B82] Parvin S, Williams CR, Jarrett SA, Garraway SM (2021) Spinal cord injury increases pro-inflammatory cytokine expression in kidney at acute and sub-chronic stages. Inflammation 44:2346–2361. 10.1007/s10753-021-01507-x 34417952 PMC8616867

[B83] Peacock JH, Nelson PG, Goldstone MW (1973) Electrophysiologic study of cultured neurons dissociated from spinal cords and dorsal root ganglia of fetal mice. Dev Biol 30:137–152. 10.1016/0012-1606(73)90053-5 4735361

[B84] Petruska JC, Cooper BY, Johnson RD, Gu JG (2000) Distribution patterns of different P2x receptor phenotypes in acutely dissociated dorsal root ganglion neurons of adult rats. Exp Brain Res 134:126–132. 10.1007/s002210000414 11026733

[B85] Pezet S, Malcangio M, Lever IJ, Perkinton MS, Thompson SW, Williams RJ, McMahon SB (2002) Noxious stimulation induces Trk receptor and downstream ERK phosphorylation in spinal dorsal horn. Mol Cell Neurosci 21:684–695. 10.1006/mcne.2002.1205 12504600

[B86] Raychaudhuri SK, Raychaudhuri SP, Weltman H, Farber EM (2001) Effect of nerve growth factor on endothelial cell biology: proliferation and adherence molecule expression on human dermal microvascular endothelial cells. Arch Dermatol Res 293:291–295. 10.1007/s004030100224 11480588

[B87] Rush AM, Bräu ME, Elliott AA, Elliott JR (1998) Electrophysiological properties of sodium current subtypes in small cells from adult rat dorsal root ganglia. J Physiol 511:771–789. 10.1111/j.1469-7793.1998.771bg.x 9714859 PMC2231151

[B88] Rush AM, Cummins TR, Waxman SG (2007) Multiple sodium channels and their roles in electrogenesis within dorsal root ganglion neurons. J Physiol 579:1–14. 10.1113/jphysiol.2006.121483 17158175 PMC2075388

[B89] Rutlin M, Ho CY, Abraira VE, Cassidy C, Bai L, Woodbury CJ, Ginty DD (2014) The cellular and molecular basis of direction selectivity of Adelta-LTMRs. Cell 159:1640–1651. 10.1016/j.cell.2014.11.038 25525881 PMC4297767

[B90] Schwab ME, Bartholdi D (1996) Degeneration and regeneration of axons in the lesioned spinal cord. Physiol Rev 76:319–370. 10.1152/physrev.1996.76.2.319 8618960

[B91] Shu XQ, Llinas A, Mendell LM (1999) Effects of trkB and trkC neurotrophin receptor agonists on thermal nociception: a behavioral and electrophysiological study. Pain 80:463–470. 10.1016/S0304-3959(99)00042-1 10342408

[B92] Siddall PJ, Loeser JD (2001) Pain following spinal cord injury. Spinal Cord 39:63–73. 10.1038/sj.sc.3101116 11402361

[B93] Sikandar S, Minett MS, Millet Q, Santana-Varela S, Lau J, Wood JN, Zhao J (2018) Brain-derived neurotrophic factor derived from sensory neurons plays a critical role in chronic pain. Brain 141:1028–1039. 10.1093/brain/awy009 29394316 PMC5888992

[B94] Silver J, Miller JH (2004) Regeneration beyond the glial scar. Nat Rev Neurosci 5:146–156. 10.1038/nrn1326 14735117

[B95] Sliwinski C, et al. (2024) Contribution of mechanoreceptors to spinal cord injury–induced mechanical allodynia. Pain 165:1336–1347. 38739766 10.1097/j.pain.0000000000003139 PMC11090032

[B96] Strickland ER, Woller SA, Hook MA, Grau JW, Miranda RC (2014) The association between spinal cord trauma-sensitive miRNAs and pain sensitivity, and their regulation by morphine. Neurochem Int 77:40–49. 10.1016/j.neuint.2014.05.005 24867772 PMC4177361

[B97] Stucky CL, DeChiara T, Lindsay RM, Yancopoulos GD, Koltzenburg M (1998) Neurotrophin 4 is required for the survival of a subclass of hair follicle receptors. J Neurosci 18:7040–7046. 10.1523/JNEUROSCI.18-17-07040.1998 9712673 PMC6792951

[B98] Stucky CL, Lewin GR (1999) Isolectin B(4)-positive and -negative nociceptors are functionally distinct. J Neurosci 19:6497–6505. 10.1523/jneurosci.19-15-06497.1999 10414978 PMC6782829

[B99] Tartaglia N, Du J, Tyler WJ, Neale E, Pozzo-Miller L, Lu B (2001) Protein synthesis-dependent and -independent regulation of hippocampal synapses by brain-derived neurotrophic factor. J Biol Chem 276:37585–37593. 10.1074/jbc.M101683200 11483592

[B100] Terracina M, Franchi J, Bonté F, Romagnoli G, Maurelli R, Failla CM, Dumas M, Marconi A, Fila C, Pincelli C (2003) Expression and function of neurotrophins and their receptors in cultured human keratinocytes. J Invest Dermatol 121:1515–1521. 10.1111/j.1523-1747.2003.12624.x 14675204

[B101] Vega JA, Vazquez E, Naves FJ, Del Valle ME, Calzada B, Represa JJ (1994) Immunohistochemical localization of the high-affinity NGF receptor (gp140-trkA) in the adult human dorsal root and sympathetic ganglia and in the nerves and sensory corpuscles supplying digital skin. Anat Rec 240:579–588. 10.1002/ar.1092400415 7879909

[B102] Vergara C, Latorre R, Marrion NV, Adelman JP (1998) Calcium-activated potassium channels. Curr Opin Neurobiol 8:321–329. 10.1016/s0959-4388(98)80056-1 9687354

[B103] Wei X, Wang L, Hua J, Jin X-H, Ji F, Peng K, Zhou B, Yang J, Meng X-W (2021) Inhibiting BDNF/TrkB.T1 receptor improves resiniferatoxin-induced postherpetic neuralgia through decreasing ASIC3 signaling in dorsal root ganglia. J Neuroinflammation 18:96. 10.1186/s12974-021-02148-5 33874962 PMC8054387

[B104] Widenfalk J, Widmer HR, Spenger C (1999) GDNF, RET and GFRα-1-3 mRNA expression in the developing human spinal cord and ganglia. Neuroreport 10:1433–1439. 10.1097/00001756-199905140-00009 10380959

[B105] Wu J, Renn CL, Faden AI, Dorsey SG (2013) TrkB.T1 contributes to neuropathic pain after spinal cord injury through regulation of cell cycle pathways. J Neurosci 33:12447–12463. 10.1523/jneurosci.0846-13.2013 23884949 PMC3721848

[B106] Yajima Y, Narita M, Usui A, Kaneko C, Miyatake M, Narita M, Yamaguchi T, Tamaki H, Wachi H, Seyama Y (2005) Direct evidence for the involvement of brain-derived neurotrophic factor in the development of a neuropathic pain-like state in mice. J Neurochem 93:584–594. 10.1111/j.1471-4159.2005.03045.x 15836617

[B107] Zhang C, Deng Y, Dai H, Zhou W, Tian J, Bing G, Zhao L (2017) Effects of dimethyl sulfoxide on the morphology and viability of primary cultured neurons and astrocytes. Brain Res Bull 128:34–39. 10.1016/j.brainresbull.2016.11.004 27836802

[B108] Zheng Y, Liu P, Bai L, Trimmer JS, Bean BP, Ginty DD (2019) Deep sequencing of somatosensory neurons reveals molecular determinants of intrinsic physiological properties. Neuron 103:598–616.e7. 10.1016/j.neuron.2019.05.039 31248728 PMC6706313

[B109] Zheng JL, Stewart RR, Gao W (1995) Neurotrophin-4/5 enhances survival of cultured spiral ganglion neurons and protects them from cisplatin neurotoxicity. J Neurosci 15:5079–5087. 10.1523/JNEUROSCI.15-07-05079.1995 7623136 PMC6577912

[B110] Zhou XF, Chie ET, Deng YS, Zhong JH, Xue Q, Rush RA, Xian CJ (1999) Injured primary sensory neurons switch phenotype for brain-derived neurotrophic factor in the rat. Neuroscience 92:841–853. 10.1016/s0306-4522(99)00027-5 10426526

[B111] Zhu YF, Ungard R, Zacal N, Huizinga JD, Henry JL, Singh G (2017) Rat model of cancer-induced bone pain: changes in nonnociceptive sensory neurons in vivo. Pain Rep 2:e603. 10.1097/pr9.0000000000000603 29392218 PMC5741358

